# An autonomous metabolic role for Spen

**DOI:** 10.1371/journal.pgen.1006859

**Published:** 2017-06-22

**Authors:** Kelsey E. Hazegh, Travis Nemkov, Angelo D’Alessandro, John D. Diller, Jenifer Monks, James L. McManaman, Kenneth L. Jones, Kirk C. Hansen, Tânia Reis

**Affiliations:** 1 Department of Medicine, Division of Endocrinology, Metabolism, and Diabetes, University of Colorado Anschutz Medical Campus, Aurora, CO United States of America; 2 Department of Biochemistry and Molecular Genetics, University of Colorado Anschutz Medical Campus, Aurora, CO United States of America; 3 Department of Obstetrics and Gynecology, Division of Reproductive Sciences, University of Colorado Anschutz Medical Campus, Aurora, CO United States of America; 4 Department of Pediatrics, Section of Hematology, Oncology, and Bone Marrow Transplant, University of Colorado Anschutz Medical Campus, Aurora, CO United States of America; University of Utah, UNITED STATES

## Abstract

Preventing obesity requires a precise balance between deposition into and mobilization from fat stores, but regulatory mechanisms are incompletely understood. *Drosophila* Split ends (Spen) is the founding member of a conserved family of RNA-binding proteins involved in transcriptional regulation and frequently mutated in human cancers. We find that manipulating Spen expression alters larval fat levels in a cell-autonomous manner. Spen-depleted larvae had defects in energy liberation from stores, including starvation sensitivity and major changes in the levels of metabolic enzymes and metabolites, particularly those involved in β-oxidation. Spenito, a small Spen family member, counteracted Spen function in fat regulation. Finally, mouse Spen and Spenito transcript levels scaled directly with body fat *in vivo*, suggesting a conserved role in fat liberation and catabolism. This study demonstrates that Spen is a key regulator of energy balance and provides a molecular context to understand the metabolic defects that arise from Spen dysfunction.

## Author summary

All animals need energy to fuel development and survive as adults. Excess energy stored as fat provides a means to endure periods when external energy is unavailable, but there is a delicate balance between accumulating sufficient fat stores and becoming obese. While the enzymes that mediate energy deposition into and mobilization from fat stores are well studied, the complex upstream regulatory pathways have not been fully worked out. We report here that two members of a conserved family of RNA-binding proteins, Spen and Nito, operate in fat storage cells in fruit fly larvae to control the expression of genes that mediate energy liberation from fat stores. Manipulating Spen or Spenito function grossly perturbs larval energy metabolism, including imbalances in the amounts of stored fats, key metabolites, and metabolic enzymes, and resulting in defects in survival under starvation conditions. Interestingly, Nito opposes Spen functions, indicative of a regulatory mechanism that helps keep energy balance in check. We find that the mouse homologs of Spen and Nito, which were known to regulate gene expression in other pathways, respond similarly to changes in body fat induced by a high-fat diet, suggesting that the balancing effect of these two proteins also prevents mammalian obesity.

## Introduction

Organisms strive to achieve homeostasis between energy intake and utilization, but also must maintain energy stores to survive when utilization exceeds intake. Demands for utilizable energy trigger hydrolysis of triglycerides stored in adipose cells to produce free fatty acids that are released into the circulatory system. Once within the energy-requiring cells, fatty acids must be conjugated first to coenzyme A and then to carnitine for transport across the inner mitochondrial membrane. During fasting, fat used for fuel is primarily derived from adipose tissue triglycerides, and the mobilization of fatty acids from triglyceride stores is a key regulatory step.

Obesity is caused by excess energy stored in the form of triglycerides (TAGs) [1]. Genetic factors dictate 40–70% of the variance in body mass index (BMI) and obesity predisposition [2–9], but understanding individual gene function in obesity is complicated by the multigenic and multi-systemic nature of the disease. *Drosophila* provides a powerful model to investigate mechanisms of energy storage and utilization [10–17]. The fat body (FB) corresponds to mammalian liver and white adipose tissue (WAT) and stores glycogen and TAGs [18]. Assessment of energy regulation during the larval stage is particularly informative, since energy is balanced between utilization (to fuel foraging behaviors and larval growth) and storage (to fuel later growth during the pupal stage) [10, 15, 19]. We previously identified 66 genes required to prevent excess fat accumulation in larvae, including many homologs of mammalian genes with established roles in energy balance [16]. In addition, we identified a class of genes for which mammalian homologs have not yet been implicated in fat regulation. These genes represent potential new directions in obesity research.

The *Drosophila split ends* (*spen*) gene is essential for viability and encodes an extremely large (>5,500 amino acids) RNA-binding protein known to regulate the transcription of key effectors of a number of signaling pathways. Spen promotes Wingless (Wg) signaling in flies and the orthologous Wnt signaling pathway in mammals [20, 21], and suppresses Notch signaling in flies and mammals [22–26]. Spen contains three RNA recognition motifs (RRMs) near its N terminus and, near the C terminus, the archetype Spen paralog and ortholog C-terminal (SPOC) domain [27]. Spenito (Nito), a much smaller (793 amino acids) Spen family member with RRMs and a SPOC domain, acts redundantly with Spen to promote Wg signaling [28], whereas during eye development it acts antagonistically to Spen [29]. Nito has additional roles in sex determination [30, 31] and neuronal function [31]. Importantly, the mammalian homologs of both Spen (SPEN/MINT/SHARP, hereafter mSpen) and Nito (Rbm15/OTT1, hereafter mNito) were recently found to be regulators of X chromosome inactivation via RRM-mediated interactions with the long, noncoding RNA (lncRNA) Xist [32–35]. In addition to activation or repression of transcription, Spen family proteins influence alternative splicing [30, 31, 36–39] and nuclear export of RNAs [36, 40, 41], and are commonly mutated in cancers [20, 42], but mechanistic details are lacking. Identification of *spen* hypomorphs in our unbiased screen for fat mutant larvae [16] represented the first evidence that Spen family proteins have a role in organismal adiposity. *spen* was independently identified in a subsequent genome-wide RNAi-based screen for increased adiposity in adult flies [43]. Mutation of the Spen homolog in *C*. *elegans*, Din-1, strongly increased stored fat, indicative of a conserved role in the regulation of fat storage [44]. However, these studies did not determine in which tissue Spen or its homologs act to control fat storage, or what defects in metabolism resulted in (or were reflected by) the accumulation of stored fat.

Here we analyze Spen and Nito function in the regulation of body fat in *Drosophila* larvae using a combination of genetic, cell biological, and biochemical approaches. We further monitor adipose tissue expression of mSpen and mNito in response to diet-induced obesity. Our results suggest a conserved RRM-mediated role for Spen homologs in the control of energy metabolism in fat storage tissues.

## Results

### Spen is necessary and sufficient to reduce fat accumulation in the fat body

**Fig 1:**
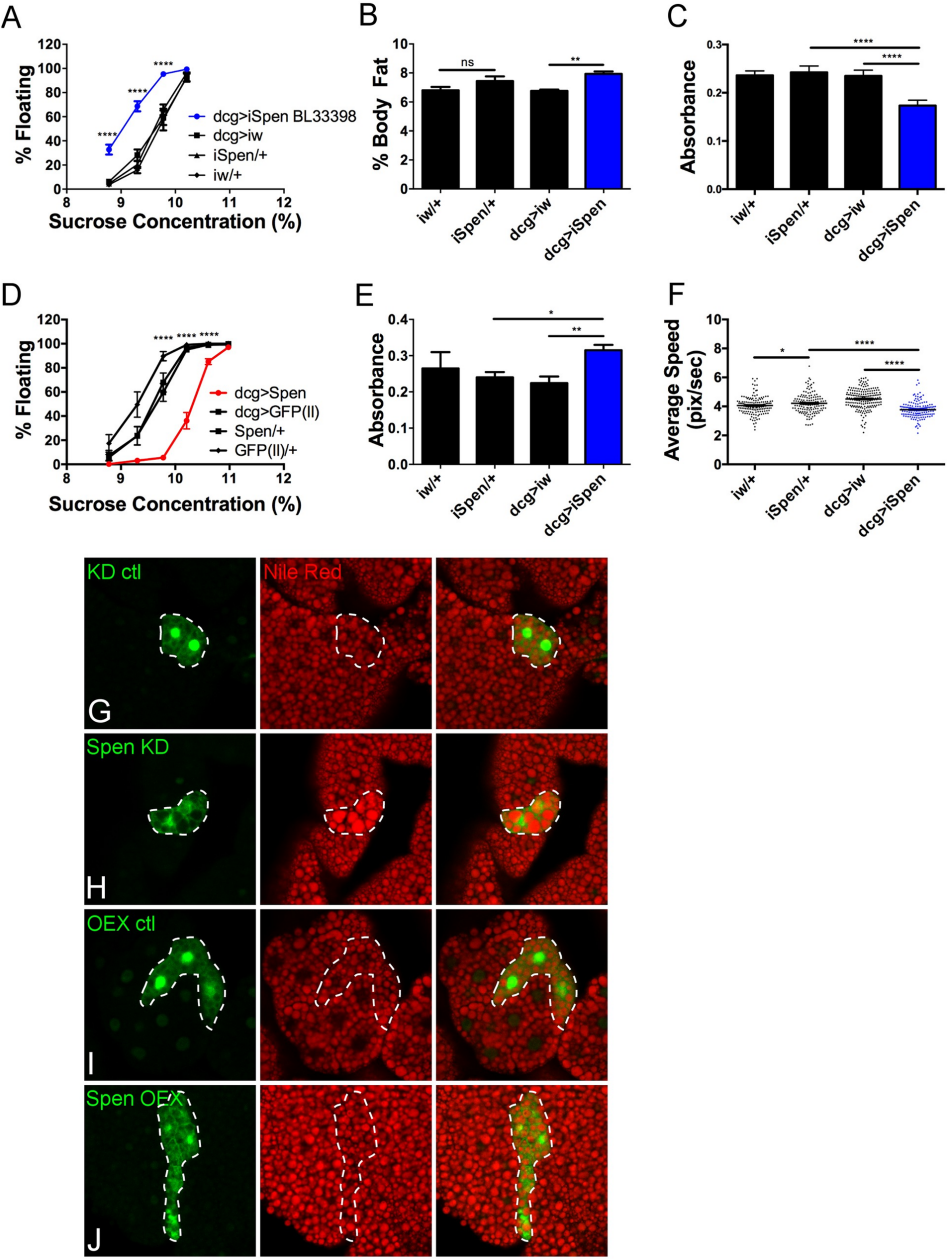
Spen autonomously decreases fat levels in the fat body. (A)Percent of floating larvae in different density solutions. FB-specific Spen KD (dcg>iSpen) compared to KD control (dcg>iw) and genetic background controls (iSpen/+ and iw/+). Fifty larvae per genotype per experimental replicate, *n* = 8 biological replicates per genotype. Error bars represent SEM. (B)Percent body fat (total neutral lipids divided by body weight) as measured by GCMS, *n* = 8. Error bars represent SEM. (C)Absorbance at 340 nm as a measure of glycogen content, *n* = 17. Error bars represent SEM. (D)As in Figure 1A, FB-specific overexpression of Spen compared to GFP overexpression and genetic backgrounds as controls. *n* = 8. Error bars represent SEM. (E)Absorbance at 530 nm as a measure of food intake, *n* = 4. Error bars represent SD. (F)Average larval speed, pixels/sec. *n* = 4 biological replicates per genotype. Error bars represent SEM. P values represent results from unpaired two-tailed *t* tests. (G-J)Larval FB tissue ectopically expressing constructs along with GFP (green). Tissues stained with the lipophilic dye Nile Red to mark neutral lipids (red). Dotted white line outlines construct-expressing clones. (G) w-RNAi, (H) Spen-RNAi, (I) UAS-GFP, (J) UAS-Spen. P values represent results from ANOVA. *P < 0.05, ** P < 0.01, ***P < 0.001, **** P < 0.0001.

Third instar (L3) larvae homozygous for a hypomorphic P-element insertion allele in the *spen* locus float in a sucrose solution in which control larvae sink, indicative of lower overall density and consistent with elevated body fat [16, 45]. Most larval fat is stored in the FB [10, 15, 19]. To test if Spen is required specifically in the FB to prevent excess fat accumulation, we measured larval density upon FB-restricted (via a dcg>GAL4 driver [46, 47]) expression of one of five distinct *spen*-targeting RNAi constructs. In all five cases, FB knockdown of Spen (dcg>iSpen, hereafter referred to as Spen KD) resulted in lower density compared to both a knockdown control (dcg>iw) and genetic background controls (iSpen/+, iw/+, and dcg/+) (Fig 1A and S1 Fig), recapitulating the whole animal mutant phenotype. Buoyancy/density correlates strongly with adiposity as assessed directly via gas chromatography coupled with mass spectrometry (GC/MS) to measure levels of neutral lipids [16]. We calculated percentage body fat in this way for the same animals tested by the buoyancy assay. KD of Spen in the FB increased body fat by ~18% (Fig 1B, mean ± SEM 8.0% ± 0.2% for Spen RNAi compared to 6.8% ± 0.1% for w RNAi control; P < 0.01 by ANOVA). Notably, although females of all genotypes stored more fat than males, for both sexes the increase in buoyancy resulting from Spen depletion was similar (mean fold change for all sucrose concentrations ± SEM, 7.9 ± 1.5 for females and 6.0 ± 1.3 for males, P = 0.34 by unpaired *t* test) (S1B and S1C Fig). Additionally, a trans-heterozygous combination of hypomorphic *spen* alleles [48] resulted in a similar density phenotype (S2A Fig). A smaller but significant decrease in density was also observed in larvae heterozygous for a null and a wildtype (WT) allele [49] (S2B Fig). Levels of glycogen, the other major form of energy stored in the FB, were also decreased in larvae when Spen was depleted (Fig 1C). FB-restricted Spen overexpression (dcg>Spen) was sufficient to drive fat depletion (Fig 1D). Our findings thus support a FB role for Spen in control of fat storage.

Both food intake and energy expenditure can influence levels of stored fat [50, 51]. To ask if changes in feeding and foraging behaviors contributed to the increase of fat levels in the Spen KD larvae, we assessed food consumption and locomotion. Spen KD in the FB increased food intake in early L3 larvae compared to controls (Fig 1E). Furthermore, pre-wandering L3 larvae showed decreased locomotor activity (Fig 1F). Both behavioral changes align with the increased stored fat in these animals. By contrast, no change in food intake or locomotion accompanied the lean phenotype resulting from FB-restricted Spen overexpression (S3A and S3B Fig), indicating that behavioral changes did not contribute to the decrease in energy stored as fat. We conclude that overexpressed Spen acts autonomously in the FB to produce these effects.

### Spen acts autonomously in fat body cells

Changes in levels of stored fat can result from changes in FB cell size or number [52, 53] or lipid droplet (LD) morphology or density [54]. To better understand the effects of Spen manipulation, we generated by flp-mediated recombination [55, 56] clones of FB cells in which Spen was either knocked down or overexpressed, surrounded by WT FB cells. GFP was co-expressed in both conditions to mark construct-expressing cells, and LDs were labeled with the lipophilic Nile Red [57] (Fig 1G–1J). Spen depletion caused significantly larger and more intensely stained LDs compared to controls (Fig 1G and 1H and S4A and S4B Fig), although FB cell size and number were unaffected (S4C and S4D Fig). FB cells overexpressing Spen were smaller, with LDs of normal size and staining intensity (Fig 1I and 1J and S4E–S4G Fig). As with Spen depletion, the number of FB cells per clone was unaffected by Spen overexpression (S4H Fig). *spen* FB mutant clones resulting from flp-mediated mitotic recombination in a heterozygous background produced significantly larger and more brightly stained LDs compared to WT clones (S2C–S2H Fig). We conclude that Spen functions autonomously in FB cells to regulate the amount of fat stored in LDs.

### Defects in energy utilization upon Spen depletion

**Fig 2:**
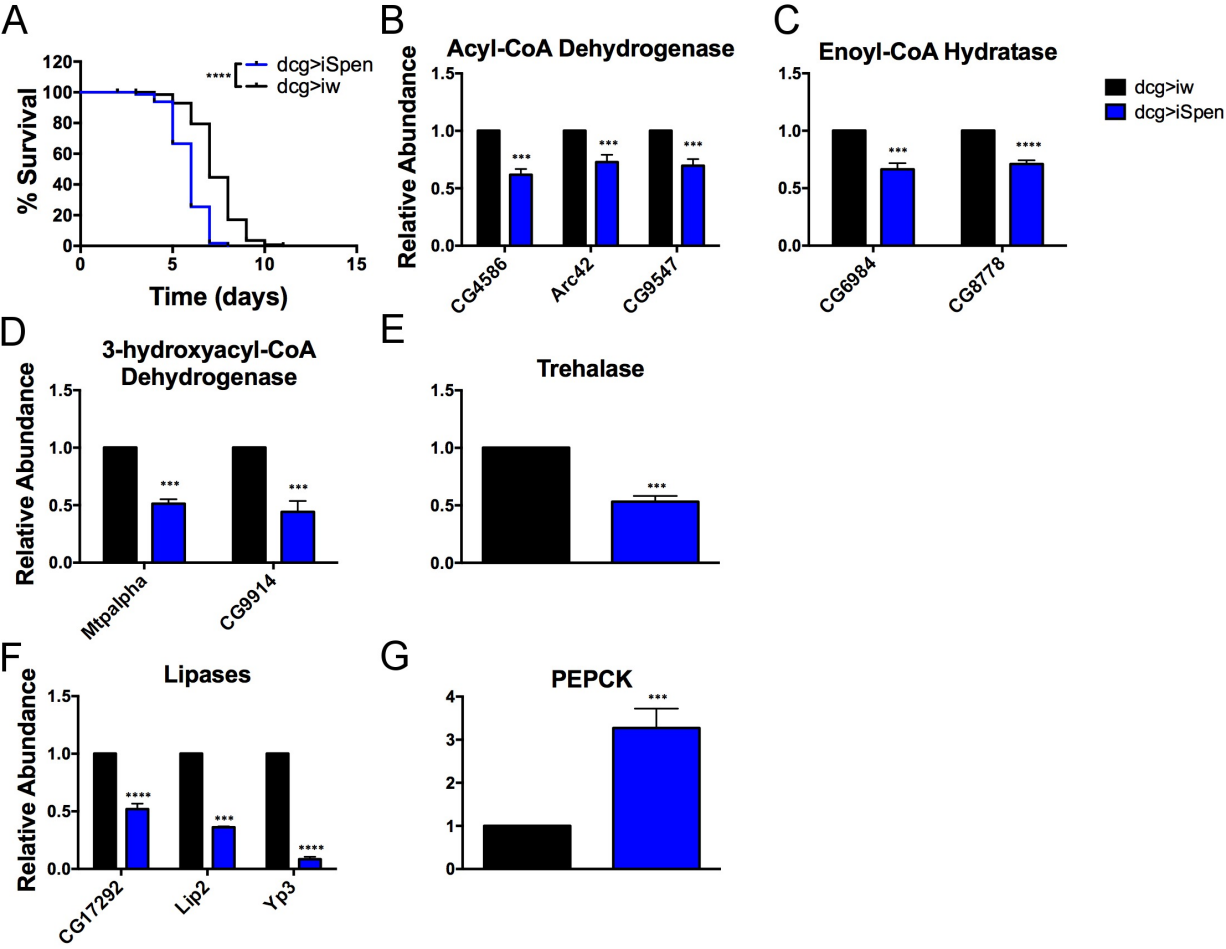
Spen regulates the breakdown of fat. (A)Survival of larvae reared in amino acid-free media. FB-specific Spen KD (dcg>iSpen) compared to KD control (dcg>iw). Fifty larvae per genotype per experimental replicate, *n* = 3. P value obtained by Log-rank test. (B-G) Gene regulation as a result of FB-specific Spen KD from RNA sequencing. Error bars represent SD. P values obtained by ANOVA. *P < 0.05, ** P < 0.01, ***P < 0.001, **** P < 0.0001.

Despite their propensity to accumulate extra fat, larvae in which Spen was depleted from the FB died more rapidly than controls when reared from hatching in a sucrose solution, i.e., deprived of fats and amino acids (Fig 2A). Spen overexpression had no effect (S3C Fig). If energy stores can be accessed normally, excess energy in the form of fat can provide a crucial advantage during starvation [16, 58]. On the other hand, the advantage is lost regardless of the abundance of stored energy if the mutant animals are unable to mobilize it. Larvae lacking Spen in the FB thus appeared to be defective in accessing energy stores and/or in extracting energy from a limited diet, and may be in a state of “perceived starvation”. Either defect could drive the overfeeding and lethargy that we observed with a regular diet (Fig 1E and 1F). Indeed, FB-specific Spen depletion also caused a one-day developmental delay (19.8 hours ± 1.3 hours as compared to dcg>iw), consistent with a dearth of available energy, although we cannot exclude other causes. These results point to a role for Spen in regulating the liberation of energy stored as fat in the FB.

### Alterations in the expression of key metabolic enzymes upon Spen manipulation point to a role in energy catabolism

Spen and its homologs influence other pathways via control of transcription [59–61]. Accordingly, we suspected that the transcript levels of key metabolic enzymes would be affected by Spen manipulation in the FB, and tested this prediction using RNA sequencing (RNAseq). 440 of the 516 genes whose levels significantly changed when Spen was KD in the FB were classified by the PANTHER system [62]. 173 (39.3%) of the classified genes were categorized as being involved in a “metabolic process”, representing the largest functional “biological process” category (followed by “cellular process”, 30.7%). We observed striking changes in transcripts encoding proteins involved in β-oxidation, a process by which fatty acids are broken down to provide acetyl-CoA for the TCA cycle. Though redundant enzymes participate in β-oxidation reactions, three key enzymes involved in this pathway were significantly downregulated in Spen KD larvae (Fig 2B–2D), namely acyl-CoA dehydrogenase, enoyl-CoA hydratase, and 3-hydroxyacyl-CoA dehydrogenase. These enzymes participate in the release of a two-carbon chain from the fatty acid. Furthermore, significant downregulation of trehalase (Fig 2E) pointed to a potential blockage in disaccharide catabolism and, as a consequence, glycolysis.

With regard to the high-fat phenotype of Spen-depleted larvae, three lipases, potentially necessary for liberating stored fat, were downregulated (Fig 2F). Additionally, PEPCK (phosphoenolpyruvate carboxykinase) was highly induced (Fig 2G), a hallmark of the starvation response [63] that fits with the predicted state of “perceived starvation” resulting from an inability to access stored fats or dietary energy. Furthermore, 39 of the 126 genes significantly upregulated in Spen-depleted FBs are induced by fasting/starvation (S1 Table and [63]), and 69 of the 390 genes significantly downregulated in Spen-depleted FBs, are downregulated upon fasting/starvation (S2 Table and [63]), providing additional evidence of the similarities between Spen depletion and starvation. These findings provide strong support for the role of Spen in modulating substrate utilization for catabolism and energy production.

### Abnormal metabolic profiles upon *Spen* manipulation

**Fig 3:**
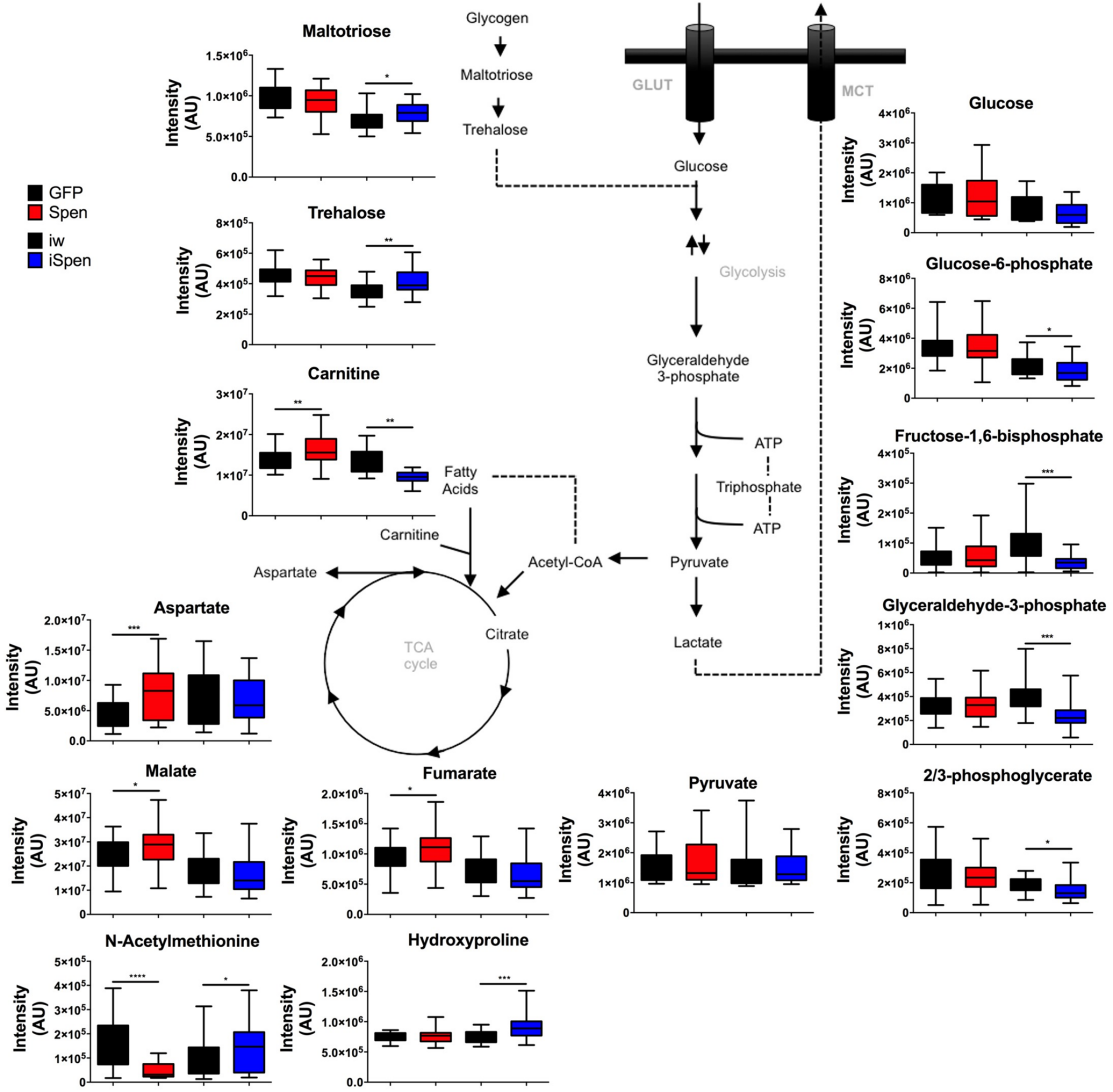
Spen manipulation results in abnormal metabolism. Quantification of selected metabolites by UHPLC-MS. Spen overexpression (Spen OEX) compared to overexpression control (GFP) and Spen KD (Spen KD) compared to KD control (iw). Ten individual larvae per biological replicate tested per genotype per 3 biological replicates, *n* = 30. Error bars represent SD. P values obtained by two-tailed *t* test. *P < 0.05, ** P < 0.01, ***P < 0.001, **** P < 0.0001.

To define at a molecular level the metabolic defects accompanying Spen manipulation, we performed Ultra-High Pressure Liquid Chromatography (UHPLC)-MS-based metabolomic analysis on larvae in which Spen was knocked down or overexpressed in the FB, along with appropriate controls for each. We monitored 178 metabolites, and found that nearly every metabolite involved in glycolysis was significantly decreased in Spen-depleted larvae (Fig 3), consistent with a depletion in these animals of key sources of usable energy, and an accumulation of molecules in which energy is stored. As in most insects, trehalose is the primary circulating sugar in *Drosophila*, and is broken down to glucose to fuel cellular processes [64, 65]. Consistent with the reduction of trehalase observed by RNAseq (1.9-fold decrease, P = 0.0009, Fig 2E), trehalose levels were significantly elevated in Spen KD larvae (Fig 3), indicating impaired conversion into glucose and thus decreased glycolytic intermediates.

In addition to defective mobilization of carbohydrate sources for energy production, we found clear defects in β-oxidation. Acyl-carnitines are key intermediates of β-oxidation that permit fatty acid transport into mitochondria [66], which is the rate-limiting step of β-oxidation. Spen KD larvae were significantly depleted of free carnitine as well as nearly every medium- and long-chain fatty acyl-carnitine (Fig 3 and S5A Fig), consistent with the observed decreases in β-oxidation enzymes (Fig 2B–2D) and suggestive of a defect in β-oxidation. Finally, the levels of many free amino acids decreased in Spen KD larvae (S5B Fig), while markers of protein catabolism n-acetylmethionine and hydroxyproline were increased in Spen KD larvae and decreased in Spen-overexpressing ones (Fig 3), consistent with increased proteolysis in response to Spen KD. Among the transcripts that increased significantly in Spen KD larvae are three predicted trypsin-family proteases (CG11529, CG31326, and CG8299, the latter increased ~80-fold) that may be good candidates to mediate elevated protein catabolism. These metabolic changes provide direct evidence of a defect in energy mobilization via catabolism of carbohydrate and lipid energy sources, and may indicate the use of amino acids as an energy source.

Importantly, FB overexpression of Spen had effects opposite to that of Spen depletion with regards to β-oxidation, including increased levels of carnitine (Fig 3). Spen overexpression did not significantly alter glycolytic metabolites or acyl-carnitine levels, although the steady state of some TCA cycle intermediates and a few amino acids were slightly elevated (Fig 3 and S5C and S5D Fig). These findings further indicate that Spen regulates fat catabolism.

### Both known domains of Spen are necessary for its function in metabolism

**Fig 4:**
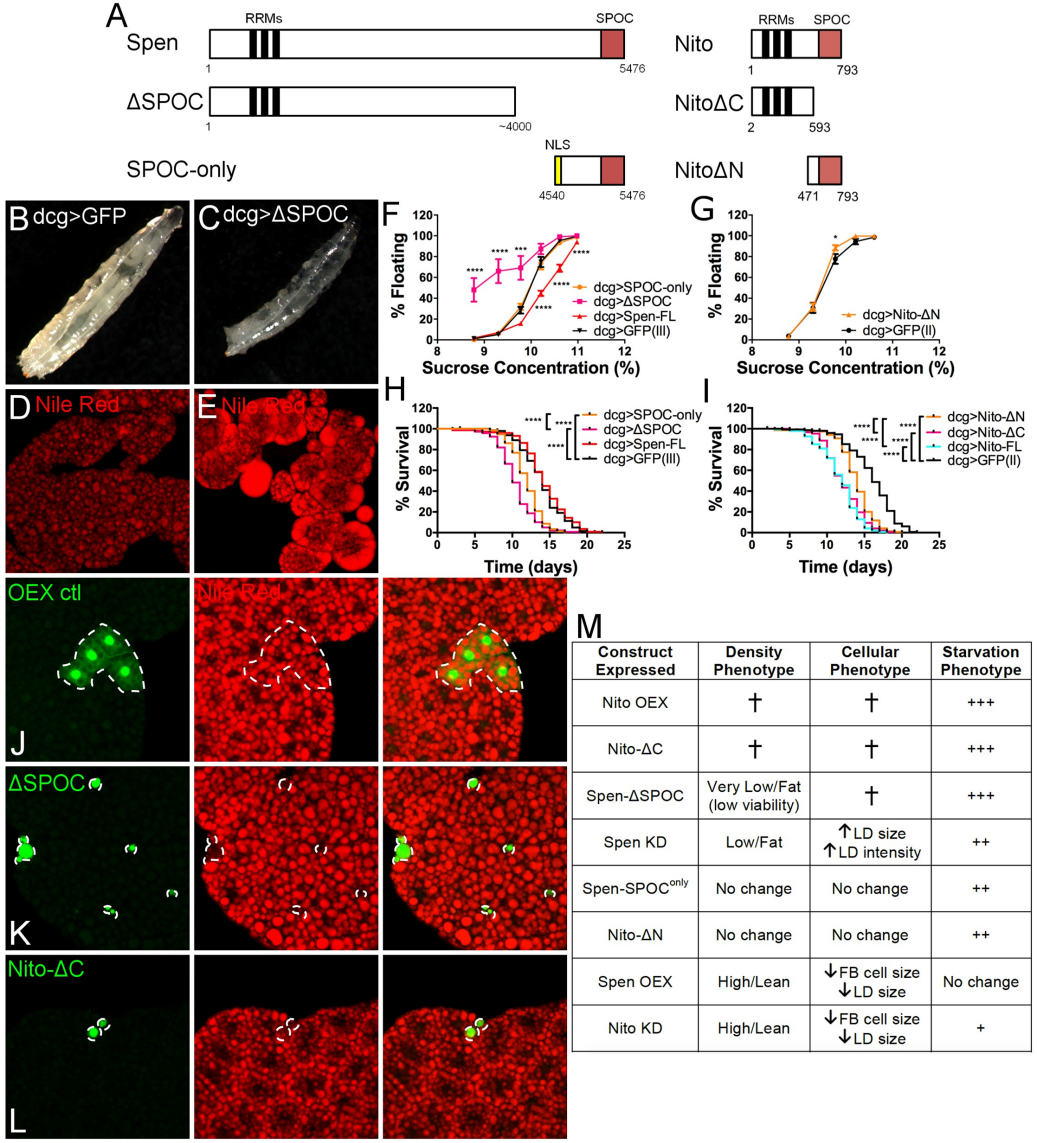
Domain analysis of Spen and Nito function in metabolism. (A)A schematic representation of Spen and Nito proteins and the location of the RNA-recognition motifs (RRMs) and SPOC domains in each. The truncation lines tested for each are pictured below. (B-C) Larvae reared at 16°C and imaged at wandering stage. (B) dcg>GFP and (C) dcg>ΔSPOC. (D-E) Larvae reared at 16°C and collected at wandering stage. Larval FB tissue stained with Nile Red. (D) dcg>GFP and (E) dcg>ΔSPOC. (F) As in Fig 1A, FB-specific expression of Spen truncation lines with UAS-GFP as a control, *n* = 8. Larvae reared at 16°C. P values obtained by ANOVA. Error bars represent SEM. (G) As in Fig 1A, FB-specific expression of Nito-ΔN with UAS-GFP as a control, *n* = 8. P values obtained by ANOVA. Error bars represent SEM. (H) As in Fig 2A, survival rate at 18°C upon amino acid starvation for FB-specific expression of Spen truncation lines with UAS-GFP as a control. P values obtained by Log-rank test. (I) As in Fig 2A, survival rate at 18°C upon amino acid starvation for FB-specific expression of Nito truncation lines with UAS-GFP as a control. P values obtained by Log-rank test. (J-L) As in Fig 1G–1J, larval FB tissue ectopically expressing constructs along with GFP (green). Tissues stained with the lipophilic dye Nile Red to mark neutral lipids (red). Dotted white line outlines construct-expressing clones. (J) UAS-GFP, (K) UAS-ΔSPOC, (L) UAS-Nito-ΔC. (M) Table summarizing the density, cellular, and starvation phenotypes for all Spen and Nito manipulations. Low density correlates with higher levels of fat while high density correlates with low levels of fat [16]. LD: Lipid droplet. FB: Fat body. † denotes early larval death (Density column) and dying cells (Cellular column). + denotes sensitivity to starvation, where more +’s indicate increased sensitivity to starvation. *P < 0.05, ** P < 0.01, ***P < 0.001, **** P < 0.0001.

Despite the extreme size of the Spen protein, only the RRMs and SPOC domain have been functionally characterized in the context of other pathways. We obtained two Spen truncation alleles, one that lacks the C-terminal region including the SPOC domain but retains the RRMs (ΔSPOC), and one that retains only the C-terminal region and lacks the RRMs (SPOC^only^) (Fig 4A). In other contexts, each allele can behave in a dominant-negative fashion. For example, expression of ΔSPOC in midline glial cells results in completely penetrant lethality [67]. Expression of SPOC^only^ with an *engrailed* driver reduces or eliminates *Senseless* expression, suggesting an absolute requirement for this domain of Spen in its regulation of Wg signaling [21]. To test for dominant negative effects in Spen regulation of fat storage, each of these alleles was overexpressed in the FB. If the truncation had no effect, we predicted that overexpression would cause a lean phenotype, as observed with Spen overexpression using two independent constructs (Figs 1D and 4F), whereas a dominant-negative effect would result in a similar phenotype to Spen depletion and elevate fat (Fig 1A).

ΔSPOC-overexpressing larvae were unable to survive at 25°C or 18°C, arresting at the L2 stage. At 16°C, where Gal4 is less active and levels of overexpression are lower [68], development was delayed by 11–13 days compared to controls (12 days 2.28 hours ± 23.3 hours as compared to dcg>GFP) and only 5–10% of larvae survived to L3. Although we cannot exclude a neomorphic effect, we favor the interpretation that this developmental delay is an extreme version of the one-day delay observed upon Spen depletion, and thus is a manifestation of “perceived starvation” resulting from dominant inhibition of Spen function in catabolism. L3 larvae obtained at 16°C were tested by buoyancy and compared to larvae overexpressing GFP, SPOC^only^, or a full-length Spen construct (Spen-FL). Whereas Spen overexpression decreased larval buoyancy (Figs 1D and 4F), expression of ΔSPOC strongly increased larval buoyancy, and expression of GFP or SPOC^only^ had no effect (Fig 4F and S6A Fig). Analysis of feeding and activity showed no significant changes (S6B and S6C Fig). By staining isolated tissues of ΔSPOC larvae with Nile Red to label neutral lipids [57], we noticed a striking phenotype resulting from ΔSPOC overexpression. FBs in these larvae were almost non-existent (Fig 4B and 4C), but the FB tissue that remained stained much more brightly and contained very large LDs, some of which appeared to have “leaked” out of FB cells (Fig 4D–4E). Unlike tissues from control animals, brighter staining was also observed in the brains, imaginal discs, and guts of larvae overexpressing Spen ΔSPOC in the FB (S7 Fig). The appearance of fat deposits in tissues where fat does not normally accumulate is consistent with the elevated body fat phenotype, and is reminiscent of similar effects documented in the *Drosophila Seipin* mutant lipodystrophy model [69, 70].

Analysis of clones of FB cells expressing Spen-FL, ΔSPOC, or SPOC^only^ along with GFP revealed that, as with other full-length Spen overexpression constructs (Fig 1I and 1J and S4G Fig), Spen-FL overexpression resulted in smaller FB cells (S8A, S8B and S8I Fig). While LD intensity was unchanged (S8F Fig), this particular Spen-FL transgene also decreased LD size (S8E Fig), a stronger phenotype than observed with the Spen-OEX transgene (Fig 1I and 1J and S4E Fig). SPOC^only^ overexpression resulted in normally-sized FB cells with no significant changes in LD or cell size or morphology (S8A, S8C and S8G–S8J Fig), indicating that the SPOC domain is required for the ability of Spen to deplete stored fat when overexpressed. ΔSPOC overexpression, on the other hand, caused a striking phenotype suggestive of catastrophic defects in metabolism. Specifically, many of the clones consisted of a few extremely small cells containing nuclei (marked with strong GFP signal) and little else (Fig 4J and 4K). ΔSPOC overexpression may cause pleotropic defects, including cell death. However, considering that similar effects have been previously documented for FB cells during starvation [71–73], we favor a model in which the SPOC domain is required for normal Spen function in fat regulation and RRMs alone sequester crucial factors in a non-functional manner. Hence, overexpressing a version of Spen harboring the RRMs but lacking the SPOC domain perturbs the ability of full-length Spen to interact with such factors and carry out its normal function(s).

FB overexpression of full-length Spen had no effect on survival during starvation (Fig 4H). Both ΔSPOC and SPOC^only^ were significantly more sensitive to starvation than controls (Fig 4H), very similar to Spen KD, although the ΔSPOC effect was far stronger. The ability of SPOC^only^ overexpression to dominantly curtail survival during starvation contrasts with the lack of observed effects on buoyancy or LD appearance in FB cells, and suggests that the roles for Spen in fat storage and the starvation response are not strictly coupled. Phenotypes of all Spen truncation lines are summarized in Fig 4M.

### Nito antagonizes Spen function in metabolism

**Fig 5:**
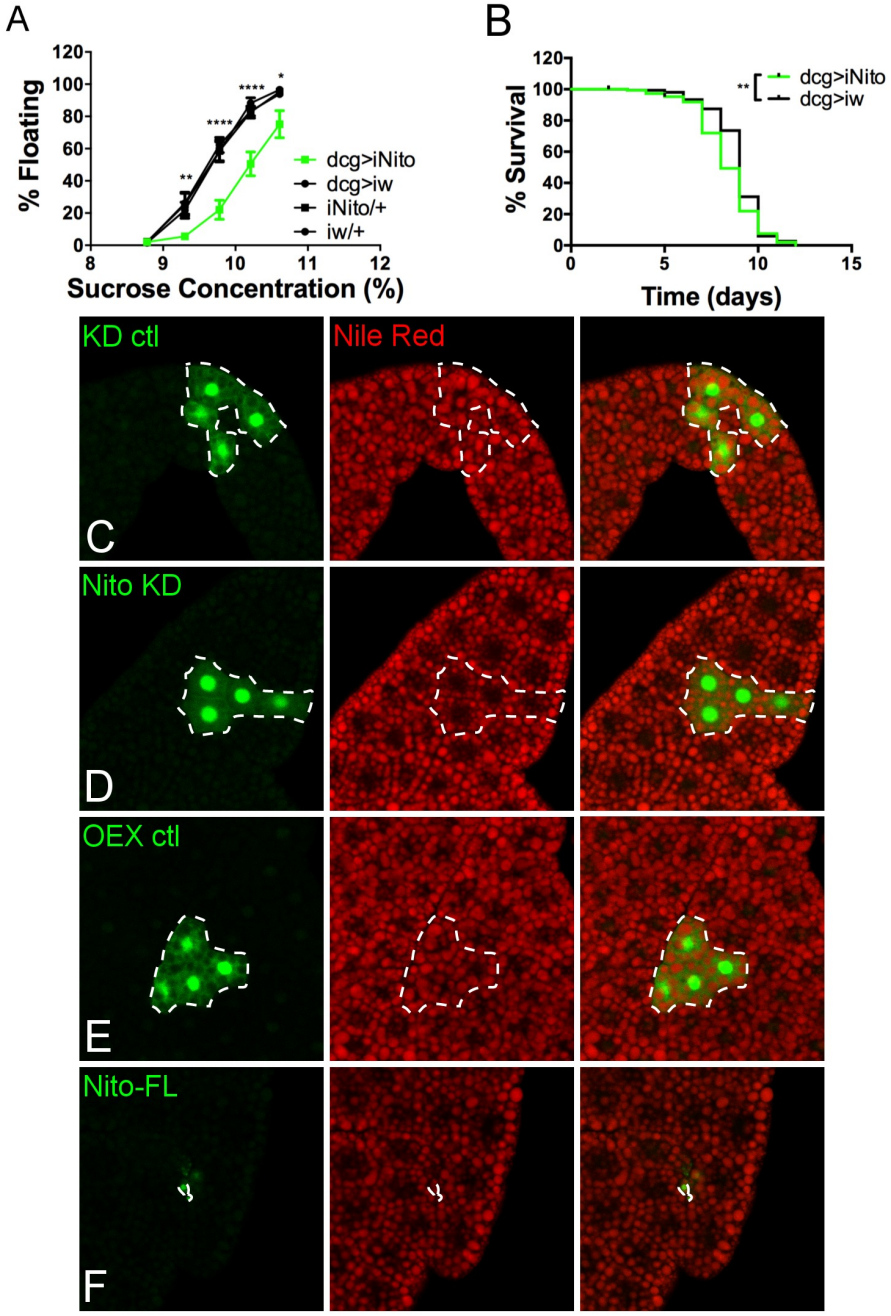
Nito autonomously promotes fat accumulation in the fat body. (A)As in Fig 1A, FB-specific Nito KD (dcg>iNito) compared to KD control (dcg>iw) and genetic background controls (iNito/+ and iw/+), *n* = 8. P values obtained by ANOVA. Error bars represent SEM. (B)As in Fig 2A, survival rate at 25°C upon amino acid starvation for FB-specific Nito KD (dcg>iNito) compared to KD control (dcg>iw). P value obtained by Log-rank test. (C-F) As in Fig 1G–1J, larval FB tissue ectopically expressing constructs along with GFP (green). Tissues stained with the lipophilic dye Nile Red to mark neutral lipids (red). Dotted white line outlines construct-expressing clones. (C) w-RNAi, (D) Nito-RNAi, (E) UAS-GFP, (F) UAS-Nito-FL. *P < 0.05, ** P < 0.01, ***P < 0.001, **** P < 0.0001.

In other pathways, Spen and Nito function either redundantly (e.g. Wg signaling [28]) or antagonistically (e.g. EGFR pathway during eye development [29]). To determine the relationship between the two Spen family members in fat regulation, we first depleted Nito from the FB and tested buoyancy. Nito depletion caused a lean phenotype (Fig 5A), similar to Spen overexpression. Introducing one copy of a Nito null allele [30] caused a very slight lean phenotype (S2A Fig) that was lost with further outcrossing (S2B Fig), hence in the absence of unknown background modifiers Nito is haplosufficient to promote normal fat storage. FB clones in which Nito was depleted had modestly smaller cells and lipid droplets, consistent with the observed lean phenotype (Fig 5C and 5D and S9A and S9C Fig). Cell number and LD intensity were not affected (S9B and S9D Fig). To ask if excess Nito inhibits Spen, we overexpressed full-length Nito in the FB. Larvae were unable to complete development even when reared at 16°C, a phenotype reminiscent of the developmental delays observed upon Spen depletion or overexpression of Spen-ΔSPOC. Full-length Nito overexpression produced clones that consisted of tiny cells in which only the nucleus was discernable (Fig 5E and 5F), similar to the effects of Spen-ΔSPOC. While we cannot at this time rule out effects on cell survival factors unrelated to metabolism, this phenotype is consistent with FB cell death due to starvation [71–73]. Finally, we asked if Nito depletion or overexpression affected sensitivity to starvation. Nito KD larvae died slightly earlier than controls (Fig 5B), as would be expected for lean animals with fewer fat stores to draw upon. Overexpression of full-length Nito caused premature death under sucrose-only conditions (Fig 4I), consistent with defects in utilization of energy from stores and/or imbalanced diets.

The N-terminal RRMs were required for these effects of Nito overexpression, as larvae overexpressing an N-terminally truncated version (Nito-ΔN) developed normally at all temperatures and were indistinguishable from controls with regard to buoyancy or other metabolic behaviors (Fig 4A, 4G and 4M and S6D–S6F Fig). On the other hand, overexpression of Nito-ΔC, which retains the RRMs but lacks the SPOC domain, caused L2 arrest regardless of temperature. Nito-ΔN overexpression did not affect lipid storage, cell size, or cell number (S8A, S8D and S8K–S8N Fig). In striking contrast, expression of a Nito-ΔC construct lacking the SPOC domain phenocopied overexpression of full-length Nito, with the majority of clones containing tiny cells (Figs 4L, 4M and 5F). Importantly, the lack of phenotypes resulting from Nito-ΔN did not reflect a failure to localize to the nucleus, as both Nito truncations localize appropriately [29]. Nito-ΔC-overexpressing larvae were sensitive to starvation, similar to the effects of full-length Nito (Fig 4I and 4M). Overexpression of Nito-ΔN caused starvation sensitivity that was milder than what we observed for full-length Nito or Nito-ΔC (Fig 4I), analogous to the effects of Spen-SPOC^only^ overexpression (Fig 4H, 4M). Taken together, these data support a model wherein Nito antagonizes Spen function in catabolism of stored energy in a mechanism that requires both the RRMs and SPOC domain, with SPOC-less Nito RRMs able to act in a potent dominant-negative manner.

### Levels of mSpen and mNito directly correlate with fat levels in mammalian adipose tissue

**Fig 6:**
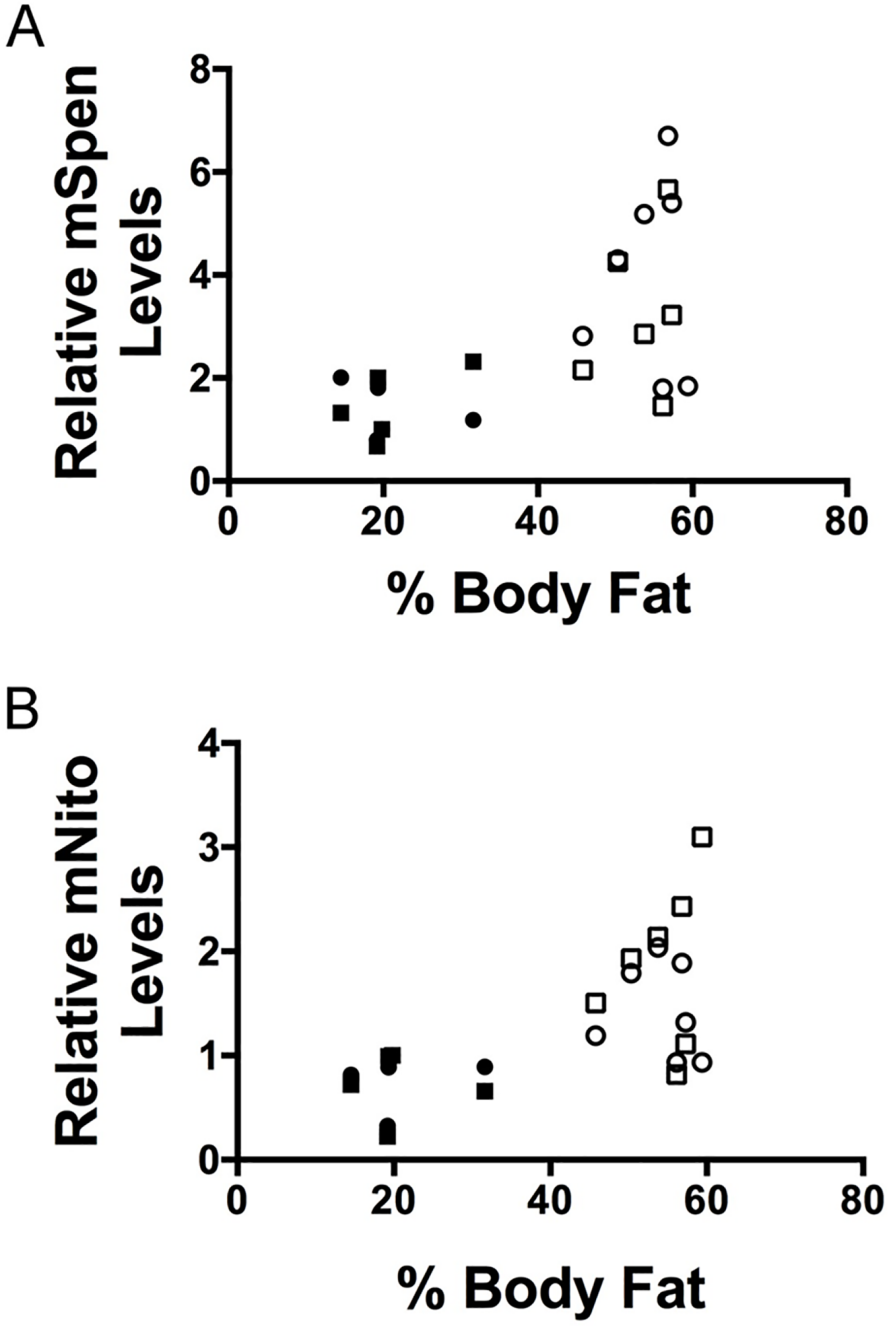
Spen and Nito transcript levels are modulated by body fat levels in mouse adipose tissue. (A)mSpen levels with respect to percent body fat in mice fed either a normal chow (solid shapes, *n* = 5) or HFD (open shapes, *n* = 7). Two technical replicates per sample (circles vs. squares). P value and correlation coefficient obtained by unpaired two-tailed *t* test from the average of the technical repeats. P < 0.05, r = 0.65. (B)As in A, mNito levels with respect to percent body fat in mice. P < 0.01, r = 0.74.

If mSpen and/or mNito function is important in preventing excess fat accumulation in mammals, we predicted that driving fat accumulation via a high-fat diet (HFD) might trigger changes in the expression of these genes in mice. For individual mice fed either normal chow or a HFD for 30 weeks, we measured both body fat percentage (mass of isolated white adipose tissue (WAT) divided by body mass) and, via RT-qPCR, mSpen or mNito transcript levels in the isolated uterine WAT. The HFD increased body fat by ~2.6-fold on average (54.2 ± 1.8%, n = 7 for HFD compared to 20.9 ± 2.9%, n = 5 for normal chow, unpaired t test P < 0.0001). Strikingly, both mSpen and mNito transcript levels (normalized to levels of 4 housekeeping genes) correlated strongly with body fat percentage (Fig 6, R = 0.65, P < 0.05 for mSpen and R = 0.74, P < 0.01 for mNito by unpaired two-tailed t test). While further studies will be required to determine how changes in mSpen and mNito expression in animals made obese by a HFD reflect the normal functions of these proteins, we take these data as evidence that Spen and Nito functions in fat storage are conserved from flies to mammals.

## Discussion

Our work provides the first detailed investigation of a fat regulatory role for Spen in any organism, and the first evidence that Nito also functions in this process. Spen depletion in the FB drastically increased stored fat (Fig 1A and 1B). Spen has been implicated in multiple pathways involved in endocrine signaling, including Notch [49, 74], Wingless [21], and nuclear receptor signaling [44, 61, 75]. We find it unlikely that nuclear receptor pathways are relevant to the fat regulatory role we define, because we did not observe upon Spen depletion or overexpression consistent changes in the expression of genes that are targets of those pathways. Furthermore, the lack of phenotypes involving fat storage *per se* upon overexpression of Spen-SPOC^only^ (Fig 4F) argues against a role for Wg signaling, in which the same construct has potent dominant negative effects [21]. Conversely, whereas a C-terminally truncated version of mSpen has little effect on Notch signaling [23], the strong fat phenotypes resulting from Spen-ΔSPOC overexpression suggest that Spen does not regulate fat via the Notch pathway.

Notably, Spen KD larvae also exhibited behavioral changes (increased food intake, decreased locomotion) that may have contributed to the fat increase (Fig 1E and 1F). Thus, in addition to direct roles in fat accumulation within fat storage cells, Spen may be involved in a cross-talk pathway between the FB and the brain. However, we strongly support a model wherein increased food intake is instead an attempt to compensate for a condition of “perceived starvation” resulting from an inability to access energy stores. Similarly, a lack of available energy may restrict locomotion. This hypothesis is further strengthened by the observation that Spen overexpression was sufficient to deplete stored fat (Fig 1D) but did not cause opposing behavioral phenotypes (S3A and S3B Fig).

Mosaic analysis confirmed an autonomous role for Spen in FB cells. Spen KD in clones throughout the FB showed a striking increase in LD size (Fig 1H and S4A Fig). Larger LDs normally have lower surface tension, and the stored fat is easier to access [76]. LD remodeling in WT animals is a highly regulated process involving specific factors, some of which were identified in a genome-wide RNAi screen in cultured *Drosophila* S2 cells [14, 77, 78]. Notably, our RNAseq data revealed that the products of several LD-regulating genes were significantly altered by Spen depletion, including l(2)01289 (~7-fold decreased, P < 0.0001 by unpaired two-tailed t test), CG3887 (1.3-fold decreased, P = 0.001), and eIF3-S9 (1.5-fold increased, P = 0.0008). While it is unclear if these changes are direct effects of Spen depletion, they may explain why LDs in Spen KD larvae are large yet apparently inaccessible, resulting in starvation sensitivity.

Consistent with the observed changes in FB cell and LD morphology and starvation sensitivity, changes in metabolites and gene expression in Spen KD larvae pointed to a drastic defect in lipid catabolism. Defects in β-oxidation were the most obvious, in part because the opposite effects were observed upon FB-restricted Spen overexpression. Spen depletion led to a decrease in the levels of free and acyl-conjugated carnitine, as well as of transcripts of three of the four enzymes necessary to break down acyl-carnitines into free fatty acids (Fig 2B–2D and Fig 3). Three lipases were also downregulated (Fig 2F), which likely further contributes to an inability to convert energy stored as TAGs into usable forms. While an apparent upregulation of gluconeogenesis is evident, as supported by alterations in aspartate (Fig 3) and PEPCK expression (Fig 2G), these processes may be unable to completely compensate for decreased trehalose utilization, and these defects may contribute to the lethargy phenotype resulting from Spen KD. Consequently, surviving the loss of Spen may require breakdown of protein into free amino acids in order to anaplerotically replenish the TCA cycle, consistent with changes in expression of proteases, the observed decrease in many free amino acids (S5B Fig), as well as increases in protein catabolism and collagen turnover markers (N-acetylmethionine and hydroxyproline) (Fig 3). Of note, sustained proteolysis is a marker of aging and inflammation, a phenotype that has been previously associated with decreased locomotion in human and mouse models of physical activity, suggesting potential future ramifications of Spen’s role in metabolism with respect to aging/inflammation research [79]. Finally, the observed decrease in glycogen levels upon Spen KD (Fig 1C) supports a model wherein glycogen is used as a carbohydrate source (in lieu of decreased levels of trehalose (Fig 2E and Fig 3)) to fuel glycolysis. The overall metabolic defects we describe are distinctly different from what has been observed upon manipulation of other fat regulators (e.g. *Sir2* [16]), suggesting that Spen operates in a previously undescribed pathway.

Our results with Spen and Nito truncations provide additional mechanistic insight into how these proteins function in fat regulation. Overexpressing Spen-ΔSPOC reversed the phenotype of full-length Spen overexpression, and instead resulted in similar phenotypes to Spen depletion. Nito-ΔC overexpression had the same effects: larvae arrested development and FB clones mimicked starvation even when dietary nutrients were abundant. Overexpression of the Spen-SPOC^only^ construct had no effect on FB cells, as was the case for Nito-ΔN. Thus only Spen harboring the RRMs and the SPOC domain was able to promote fat depletion when overexpressed. Conversely, only truncated forms of Spen or Nito that retain the RRMs dominantly perturbed both FB cell viability and organismal resistance to starvation.

Recent studies of X chromosome inactivation found that mSpen RRMs mediate binding to the lncRNA Xist [32–35]. Rbm15 (mNito) also binds Xist [32, 33], and is required for N^6^-methyladenosine (m^6^A) modification of that lncRNA, which is in turn required for its ability to repress X chromosome transcription [80]. Nito is a subunit of the *Drosophila* m^6^A methyltransferase complex and is required for RNA binding by that complex; Nito knockdown severely decreases global m^6^A modification of mRNA [31]. Interestingly, the m^6^A demethylase FTO/ALKBH9 was the first human obesity susceptibility gene identified by genome-wide association studies [81–83], but the relevant nucleic acid target(s) remain unknown. Our work provides the first hint that an RNA bound by Spen and/or Nito may be a key FTO substrate.

**Fig 7:**
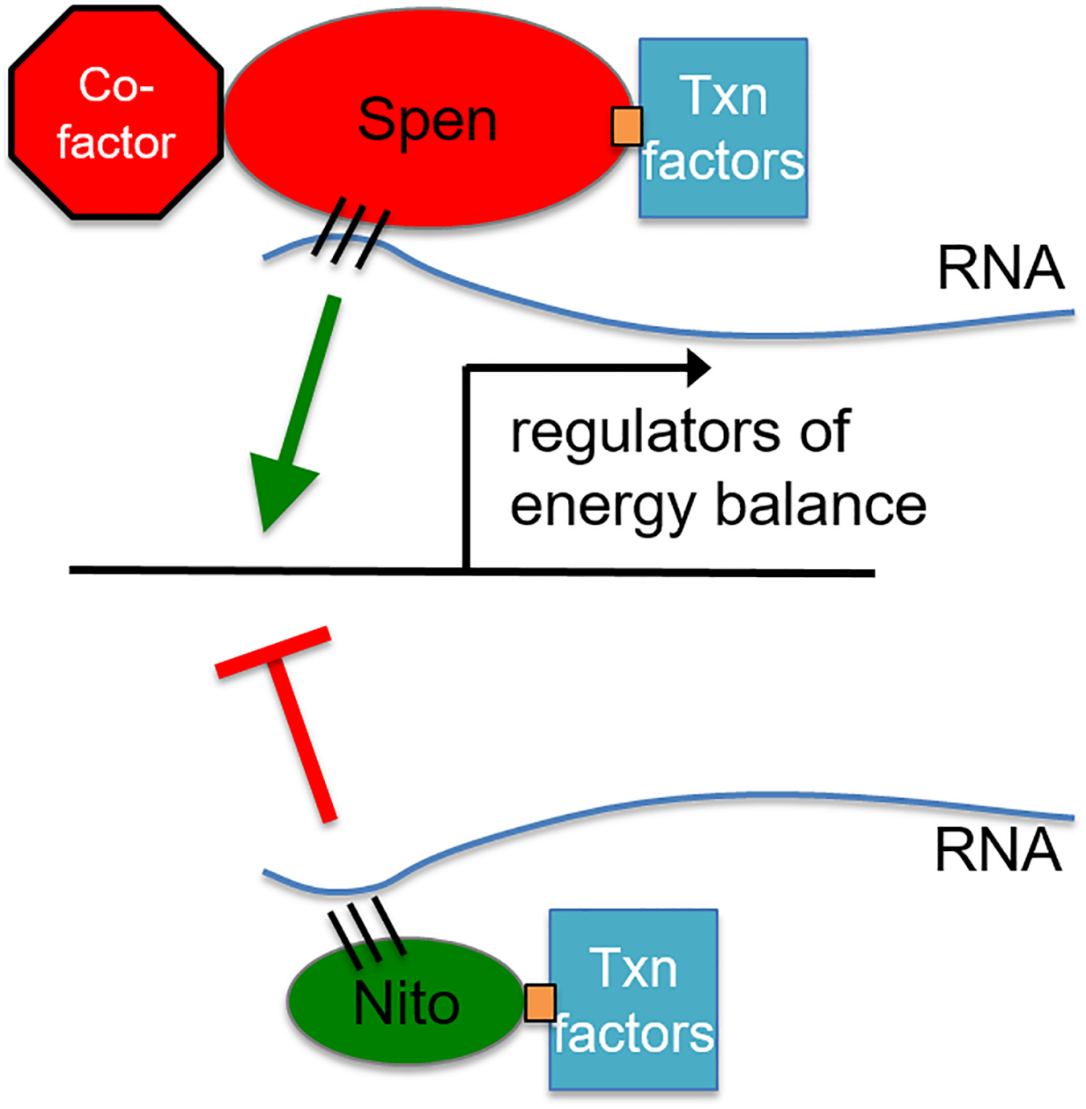
Model: Spen family members counter-regulate metabolism. Our model predicts that Spen and Nito bind the same or similar RNAs via the RRMs as well as transcription factors via the SPOC domain. Spen acts to activate enzymes key for the mobilization of energy stored as fat while Nito antagonizes this function. Spen may achieve this activation by binding additional factors in the uncharacterized region between the RRMs and SPOC domain not found in Nito.

These findings lead us to propose a model for Spen and Nito function in the regulation of fat storage (Fig 7). Spen binds via its RRMs to one or more RNAs and, via recruitment of other factors, promotes the expression of enzymes key for mobilization of energy stored as fat (e.g. lipases). The mechanism of activation may be direct or indirect, and via alternative splicing, activation/repression of transcription, or effects on RNA stability and/or translation. Moreover, RNA binding partners may be mRNA or non-coding RNA. Future work will be required to make these distinctions. We propose that the Spen SPOC domain is critical for this function, but undefined domains in between the N-terminal RRMs and C-terminal SPOC domain are also important, and these are not shared with Nito. We propose that Nito binds via its RRMs the same or a largely overlapping set of RNAs, and also recruits additional factors via its SPOC domains, but–either because it fails to recruit specific factors recruited by Spen, or because it recruits other factors not recruited by Spen—Nito ultimately inhibits/represses the same energy-storage-mobilizing enzymes that are activated by Spen (Fig 7). Overexpressed Spen or Nito fragments containing RRMs sequester target RNAs away from endogenous full-length Spen and the other effectors of fat storage control. Finally, our findings in mouse adipose tissue that mSpen and mNito both increase in expression when a HFD drives fat accumulation lead us to believe that in WT animals Nito acts as a counterbalance to Spen in order to fine-tune fat storage. Future studies delving into more mechanistic details may lead to treatments for obesity and related metabolic disorders that result from perturbation of the pathway that we elucidate here.

## Materials and methods

### Fly strains and husbandry

W^1118^ (3605), w; dcg>Gal4 (7011), y^1^ sc v^1^; +; UAS-Spen RNAi (33398), y^1^ v^1^; UAS-Spen RNAi (50529), y^1^ sc v^1^; +; UAS-Nito RNAi (34848), y^1^ v^1^; +; UAS-w RNAi (28980), y^1^ w; UAS-Spen (20756), w; UAS-GFP (9331), w; +; UAS-GFP (9330), Spen^14O1^ (5808), Spen^16H1^ (5809), Spen^3^ (8735), and Spen^5^ (8734) were obtained from the Bloomington stock center. Spen^14O1^ is an hypomorphic allele while Spen^16H1^ is a null [48]. Spen^3^ and Spen^5^ are null alleles, caused by small deletions in the Spen locus leading to truncations of the protein [49]. w; +; UAS Spen RNAi (48848), w; UAS Spen RNAi (45943), w; UAS Spen RNAi (108828), and w; UAS w RNAi (30033) we obtained from the Vienna Drosophila Resource Center (predicted off-targets in Table 1). w; UAS-SPOC^only^, w; UAS-ΔSPOC, w; UAS-Spen-FL, w; UAS-Nito-ΔN, w; UAS-Nito-ΔC, and w; UAS Nito-FL were generous gifts from Ilaria Rebay and Ken Cadigan. ΔSPOC contains all but the last ~1500 amino acids of Spen [67] while SPOC^only^ contains only the last 936 amino acids of Spen as well as a nuclear localization signal [21]. Nito-ΔC contains the first 593 amino acids of Nito while Nito-ΔN contains only the last 322 amino acids of Nito [29]. Nito^1^ is a null mutant that was a generous gift from Norbert Perrimon [30]. w; act>cd2>Gal4 UAS-GFP was obtained elsewhere [55]. y^1^ sc v^1^; +; UAS-Spen RNAi (33398) was used for all Spen KD experiments excluding those explicitly stated otherwise. y^1^ w; UAS-Spen (20756) is an EP overexpression line used for overexpression experiments including the initial density assay, RNA sequencing, and metabolomics analysis. w; UAS-Spen-FL was a generous gift from Bertrand Mollereau [84] and is a full-length Spen insertion used for subsequent overexpression experiments including truncation density and starvation assays and clonal analysis. Similar results were obtained with both the Spen-EP and Spen-FL lines. Animals were reared at 25°C unless otherwise specified and fed a modified Bloomington media (with malt) containing 35g yeast per liter. Food was made fresh each week and used within the week. Eggs were collected on grape plates at 25°C and 50 first-instar larvae were transferred 22–24 hours later into a vial of food.

**Table 1:** Predicted off-targets for the RNAi lines used and references for the figures in which they were used.

RNAi Line	Figure	Predicted Off-Targets
Spen RNAi BL33398	All main figures S1A-C	None
Spen RNAi BL50529	S1D-F	None
Spen RNAi v48846	S1G-H	CG18740
Spen RNAi v49543	S1G-H	CG17834
Spen RNAi v108828	S1G-H	CG31517, CG32697
Nito RNAi BL34848	5, S9	None

### Density assay

Density assays were performed as previously described [16, 45] with 50 larvae per sample (*n* = 8 samples per genotype). For sex-specific density assays, two samples of 50 larvae each were collected, pooled, and sorted for sex prior to performing the assay. ANOVA was used to calculate statistical significance with Prism 6 software.

### Gas chromatography mass spectrometry

Ten larvae from the group tested in the buoyancy assay (including both floaters and sinkers) were collected, frozen in liquid nitrogen, and weighed as a group. Larvae were homogenized and neutral lipids were extracted and analyzed as previously described [16, 85] using a Thermo Fisher Trace 1300-ISQ GC/MS system. *n* = 8. ANOVA was used to calculate statistical significance with Prism 6 software.

### Glycogen quantification

Ten wandering third instar larvae were collected and frozen in liquid nitrogen. Larval samples were prepared using the Hexokinase (HK) Assay Kit (Sigma, St. Louis, MO) as described [86]. Briefly, animals were homogenized and heat treated. Sample was divided into two sets, one which was treated with amyloglucosidase to digest glycogen and one that was treated with PBS. These samples along with glycogen and glucose standards treated similarly were incubated for 1 hour at 37°. 100 μL HK reagent was added to each standard and sample and measured for absorbance at 340 nm in 96-well plates using a Cytation 3 plate reader (BioTek, Winooski, VT). Glycogen levels were determined by subtracting the absorbance measured for the untreated samples (basal glucose level) from the amyloglucosidase treated samples. *n* = 17. ANOVA was used to calculate statistical significance with Prism 6 software.

### Feeding assay

Thirty early L3 larvae were collected, placed on a spot of yeast paste containing 0.5% food dye FD&C Red #40 on an agar plate at 25°C and the larvae allowed to eat for 30 minutes and processed as previously described [16]. *n* = 4. ANOVA was used to calculate statistical significance with Prism 6 software.

### Activity assay

Pre-wandering L3 larvae were collected and tracked for movement as previously described [87]. *n* = 4. Two-tailed unpaired *t* test was used to calculate statistical significance with Prism 6 software.

### Mosaic analysis

Wandering third instar larvae of the genotypes *hs flp; act>cd2>gal4 UAS-GFP*, *hs flp; act>cd2>gal4 UAS-GFP UAS-Spen RNAi*, *hs flp; act>cd2>gal4 UAS-GFP UAS-Nito RNAi*, *hs flp; act>cd2>gal4 UAS-GFP UAS-w RNAi*, *hs flp; act>cd2>gal4 UAS-GFP UAS-Spen*, *hs flp; act>cd2>gal4 UAS-GFP UAS-Spen-ΔSPOC*, *hs flp; act>cd2>gal4 UAS-GFP UAS-Spen-SPOC*^*only*^, *hs flp; act>cd2>gal4 UAS-GFP UAS-Spen-FL*, *hs flp; act>cd2>gal4 UAS-GFP UAS-Nito-ΔC*, *hs flp; act>cd2>gal4 UAS-GFP UAS-Nito-ΔN*, and *hs flp; act>cd2>gal4 UAS-GFP UAS-Nito-FL* were dissected, fixed and stained with Nile Red (Invitrogen), as described in more detail elsewhere [16]. Stained tissues were imaged on a Leica TCS SP5 laser-scanning confocal microscope with LASAF software.

Mitotic clonal analysis was performed using larvae of the genotype *hs flp; FRT40A ubi>GFP / FRT40A* and *hs flp; FRT40A ubi>GFP / FRT40A Spen*^*5*^. These animals were heat shocked directly after egg deposition for 3 hours at 37°. Larvae were collected at wandering stage and dissected, fixed, and stained with Nile Red as above.

Clones were analyzed for LD size and intensity using an algorithm written for ImageJ. Briefly, all clones were outlined and region location recorded. The FB tissue boundary was selected based on threshold. Once clone and tissue boundaries were defined, LDs were automatically outlined based on intensity threshold of the LD and measured for size and average pixel intensity. LDs of each clone were then compared to the LDs from surrounding non-manipulated cells as well as to KD or OEX control clones. FB cell size was analyzed by manually outlining each cell within the clones and measuring for area. Two-tailed unpaired *t* tests were used to calculate statistical significance with Prism 6 software. FB cell number was calculated by manually counting the number of cells within each clone. ANOVA was used to calculate statistical significance with Prism 6 software.

### Starvation assay

Fifty larvae were placed in 20% sucrose/PBS and analyzed daily for survival. Dead larvae were removed immediately after scoring and the sucrose was changed daily. Log-rank test was used to calculate statistical significance with Prism 6 software.

### RNA library preparation and sequencing

Forty larval fat bodies were dissected for each genotype and total RNA was extracted using Trizol (Life Technologies) reagent following manufacturer's instructions. A total of 200–500 ng of total RNA was used to prepare the Illumina HiSeq libraries according to manufacturer’s instructions for the TruSeq Stranded mRNA Library Prep Kit. The mRNA template libraries were sequenced on the Illumina HiSeq4000 platform at the University of Colorado’s Genomics and Sequencing Core Facility using a 1x50bp format. Derived sequences were analyzed by applying a custom computational pipeline consisting of the open-source gSNAP [88], Cufflinks, and R for sequence alignment and ascertainment of differential gene expression [89–93]. Briefly, reads generated were mapped to the *Drosophila* genome by gSNAP [88], expression (FPKM) derived by Cufflinks [94], and differential expression analyzed with ANOVA in R. GO annotations were predicted using Panther 11.1 [62], Gene Ontology versions 1.2, annotation 2017-04-24.

### Metabolomics

Briefly, individual larvae (10 per sample, *n* = 3 samples per genotype) were suspended in 1 ml of methanol/acetonitrile/water (5:3:2, v/v) pre-chilled to -20°C and vortexed continuously for 30 min at 4°C. Insoluble material was removed by centrifugation at 10,000xg for 10 min at 4°C and supernatants were isolated for metabolomics analysis by UHPLC-MS. Analyses were performed as previously described [95–97] using a Vanquish UHPLC system coupled to a Q Exactive mass spectrometer (Thermo Fisher Scientific, San Jose, CA, USA. Graphs, heat maps and statistical analyses (either *t*-Test or ANOVA) were performed with GraphPad Prism 5.0 (GraphPad Software, Inc, La Jolla, CA).

### Murine analysis

All procedures involving animals were performed in accordance with published National Institutes of Health Guidelines. The University of Colorado Anschutz Medical Campus Institutional Animal Care and Use Committee approved this study and all procedures and housing conditions used to complete it. Mice were housed in facilities at the Anschutz Medical Campus’s Center for Comparative Medicine with free access to food and water for the study’s duration (22–24°C; 14:10 h light-dark cycle). Female C57BL/6 mice were bred in house. At 8 weeks of age mice either continued on chow diet (Harlan 2920xi) or were placed on a defined high fat diet (60% kcal fat; Research Diets D12492i) for 30 weeks. Body weights were collected weekly. Upon completion of the feeding experiments, determination of the body composition of each animal was performed by quantitative magnetic resonance EchoMRI-900 whole-body composition analyzer (Echo Medical Systems; Houston, TX). At termination, mice were euthanized with CO_2_, followed by heart puncture. Tissues were collected and immediately frozen in liquid N_2_. Total RNA was extracted from ~50 mg uterine adipose tissue for each mouse sample using Trizol (Life Technologies) reagent following manufacturer's instructions. RT was performed using Oligo d(T) 23 and M-MuLV Reverse transcriptase (NEB) per manufacturer's instructions. qPCR was performed using PowerUp^TM^ SYBR^®^ Green Master Mix (Applied Biosystems). Reactions were run in an Applied Biosystems Step One Plus qPCR machine. Primer sequences: mSpen: 5’-ggctctggttctctacagcg-3’ and 5’-ctccatgcagtgataaaatgcc-3’ mNito: 5’-gcactggccaaatctgaagaag-3’ and 5’-tccatcagaggcccatgtaaac-3’. mNito results were also confirmed with an independent primer pair. Two technical repeats were performed on 5–7 biological replicates (standard chow *n* = 5, HFD *n* = 7). Percent body fat was calculated by dividing the fat mass by total body weight. P value and correlation coefficient was obtained by unpaired two-tailed *t* test from the average of the technical repeats using Prism 6 software.

## Supporting information

S1 Fig Knockdown of Spen results in a low-density phenotype. (A)Percent of floating larvae in different density solutions. FB-specific Spen KD (dcg>iSpen, BL33398) as in Fig 1A with additional dcg/+ background control. Fifty larvae per genotype per experimental replicate, *n* = 8 biological replicates per genotype.(B)Percent of female only Spen KD larvae floating.(C)Percent of male only Spen KD larvae floating.(D)FB-specific Spen KD (dcg>iSpen, BL50529) with different insertion site as Spen KD in Fig 1A compared to KD control (dcg>iw).(E)Genetic background controls (iSpen/+ and iw/+) for (D).(F)As the Spen hairpin insertion site appears to result in a lean phenotype, KD animals were normalized to their genetic background.(G)As in (A), three additional independent Spen hairpin constructs (dcg>iSpen) tested in different density solutions and compared to KD control (dcg>iw).(H)Genetic background controls (iSpen/+’s and iw/+) for (G).
P value obtained by ANOVA. *P < 0.05, ** P < 0.01, ***P < 0.001, **** P < 0.0001. Error bars represent SEM.
(TIF)

S2 Fig Decreased larval density in Spen mutants. (A)Percent of floating larvae in different density solutions. Heterozygous Spen and Nito mutants compared to w^1118^ control. w^1118^ control and mutants were backcrossed to w; sco/cyo GFP for two generations. Fifty larvae per genotype per experimental replicate, *n* = 8–16 biological replicates per genotype. Error bars represent SEM.(B)As in (A), additional heterozygous Spen null mutants and Nito mutant compared to w^1118^ control. w^1118^ control and mutants were backcrossed to w; sco/cyo GFP for six generations.(C-D) Larval FB tissue from heterozygous Spen^5^ mutant animals expressing WT (bright green) or fully mutant (no green) clonally. Tissues stained with the lipophilic dye Nile Red to mark neutral lipids (red). (C) WT control, (D) Spen^5^. Error bars represent SEM.(E-F) Lipid droplet (LD) size in WT control (E) or Spen^5^ (F) clones. GFP/GFP represents fully WT cells, GFP/WT or Spen^5^ represents heterozygous cells, and WT/WT or Spen^5^/Spen^5^ represents recombined mutant (or control) cells. (E) GFP/GFP *n* = 143, WT/WT *n* = 191, (F) GFP/GFP *n* = 175, Spen^5^/Spen^5^ *n* = 184. Error bars represent SEM. P value obtained by unpaired two-tailed *t* tests.(G-H) LD intensity in WT control (G) or Spen^5^ (H) clones. (E) GFP/GFP *n* = 143, WT/WT *n* = 191, (F) GFP/GFP *n* = 175, Spen^5^/Spen^5^ *n* = 184. Error bars represent SEM. P value obtained by unpaired two-tailed *t* tests.
*P < 0.05, ** P < 0.01, ***P < 0.001, **** P < 0.0001.
(TIF)

S3 Fig Spen overexpression does not alter behavior or starvation response. (A)Absorbance at 530 nm as a measure of food intake for Spen overexpression (dcg>Spen) compared to overexpression control (dcg>GFP) and genetic background controls (Spen/+ and GFP/+), *n* = 4. P value obtained by ANOVA. Error bars represent SD.(B)Average larval speed, pixels/sec. *n* = 4. P value obtained by unpaired two-tailed *t* test. Error bars represent SEM.(C)Larvae reared in amino acid-free media and tracked for survival. Fifty larvae per genotype per experimental replicate, *n* = 3. P value obtained by Log-rank test.
*P < 0.05, ** P < 0.01, ***P < 0.001, **** P < 0.0001.
(TIF)

S4 Fig Spen autonomously regulates fat levels in the fat body. (A)Lipid droplet (LD) size in Spen KD (iSpen) clones or KD control (iw) clones compared to non-clone cells (denoted as background). Spen KD *n* = 91. W KD *n* = 140.(B)LD intensity in Spen KD or KD control clones compared to non-clone cells.(C)FB cell size of Spen KD and KD control clones.(D)Percentage of numbers of cells within each clone of Spen KD compared to KD control. P value obtained by ANOVA.(E)As in (A), LD size in Spen OEX clones compared to OEX control (GFP) clones and non-clone cells (denoted as background). Spen OEX *n* = 175. GFP *n* = 220.(F)As in (B), LD intensity in Spen OEX clones compared to OEX control clones and non-clone cells.(G)As in (C), FB cell size of Spen OEX clones compared to OEX control clones.(H)As in (D), percentage of number of cells per clone of Spen OEX compared to OEX control.
Error bars represent SEM. P values obtained by unpaired two-tailed *t* test. * P < 0.05, ** P < 0.01, ***P < 0.001, **** P < 0.0001.
(TIF)

S5 Fig Spen depletion or overexpression alters acyl-carnitine and amino acid levels. (A)Levels of acyl-carnitines in Spen KD larvae (dcg>iSpen) compared to KD control (dcg>iw) as determined by UHPLC.(B)Levels of amino acids in Spen KD larvae compared to w KD as determined by UHPLC.(C)As in (A), Spen OEX (dcg>Spen) compared to OEX control (dcg>GFP).(D)As in (B), Spen OEX compared to OEX control.
P values obtained by unpaired two-tailed *t* test. * P < 0.05, ** P < 0.01, ***P < 0.001, **** P < 0.0001. Error bars represent SD.
(TIF)

S6 Fig Ectopic expression of truncated Spen or Nito does not alter behavior. (A)Percent of floating larvae in different density solutions. Genetic background controls for Fig 3F. Fifty larvae per genotype per experimental replicate, *n* = 8 biological replicates per genotype. P values obtained by ANOVA. Error bars represent SEM.(B and E) Absorbance at 530 nm as a measure of food intake, *n* = 4. (B) FB specific OEX of Spen-FL (dcg>Spen-FL) and SPOConly (dcg>SPOC-only) compared to OEX control (dcg>GFP) and genetic background controls (Spen-FL/+, SPOC-only/+, and GFP/+), (E) FB-specific OEX of Nito-ΔN (dcg>Nito-ΔN) compared to OEX control (dcg>GFP) and genetic background controls (Nito-ΔN /+ and GFP/+). P values obtained by ANOVA. Error bars represent SD.(C and F) Average larval speed, pixels/sec. *n* = 4. P values obtained by unpaired two-tailed t test. Error bars represent SEM.(D)As in (A), genetic background controls for Fig 3G.
*P < 0.05, ** P < 0.01, ***P < 0.001, **** P < 0.0001.
(TIF)

S7 Fig Expression of ΔSPOC results in inappropriate fat storage in other organs. (A and B) Larvae reared at 16°C and collected at wandering stage. (A) dcg>GFP and (B) dcg>ΔSPOC larval brains stained with Nile Red.(C and D) Imaginal discs.(E and F) Guts.(G and H) Salivary Glands.
(TIF)

S8 Fig Ectopic expression of truncated Spen or Nito does not alter FB cell or LD morphology. (A-D) Larval FB tissue ectopically expressing constructs along with GFP (green). Tissues stained with the lipophilic dye Nile Red to mark neutral lipids (red). Dotted white line outlines construct-expressing clones. (A) UAS-GFP (as in Fig 4J), (B) UAS-Spen-FL, (C) UAS-Spen-SPOC^only^, (D) UAS-Nito-ΔN. Clones were obtained without heat shock induction of flp but instead from “leaky” flp expression during FB development.(E)Lipid droplet (LD) size in Spen-FL or OEX control (GFP) clones compared to non-clone cells (denoted as background). Spen-FL *n* = 161. GFP *n* = 147.(F)LD intensity in Spen-FL or control clones compared to non-clone cells.(G)As in (E), LD size in Spen-SPOC^only^ clones compared to control clones and non-clone cells. Spen-SPOC^only^ *n* = 117. GFP *n* = 147.(H)As in (F), LD intensity in Spen-SPOC^only^ clones compared to control clones and non-clone cells.(I)FB cell size of Spen-FL, Spen-SPOC^only^, and control clones.(J)Percentage of numbers of cells within each clone of Spen-FL and Spen-SPOC^only^ compared to control. P value obtained by ANOVA.(K)As in (E), LD size in Nito-ΔN clones compared to control clones and non-clone cells. Nito-ΔN *n* = 136. GFP *n* = 158.(L)As in (F), LD intensity in Nito-ΔN clones compared to control clones and non-clone cells.(M)As in (I), FB cell size of Nito-ΔN clones and control clones.(N)As in (J), percentage of number of cells within each clone of Nito-ΔN compared to control.
Error bars represent SEM. P values obtained by unpaired two-tailed *t* test. * P < 0.05, ** P < 0.01, ***P < 0.001, **** P < 0.0001.
(TIF)

S9 Fig Nito autonomously regulates fat levels in the FB. (A)Lipid droplet (LD) size in Nito KD (iNito) or control (iw) clones compared to non-clone cells (denoted as background). Nito KD *n* = 204. w KD *n* = 241.(B)LD intensity in Nito KD or control clones compared to non-clone cells.(C)FB cell size of Nito KD and control clones.(D)Percentage of numbers of cells within each clone of Nito KD compared to control. P value obtained by ANOVA.
Error bars represent SEM. P values obtained by unpaired two-tailed *t* test. *P < 0.05, ** P < 0.01, ***P < 0.001, **** P < 0.0001.
(TIF)

S1 Table Genes that are upregulated upon both Spen depletion in the FB and under starvation conditions [63]. (DOCX)

S2 Table Genes that are downregulated upon both Spen depletion in the FB and under starvation conditions [63]. (DOCX)

S1 Data Levels of 178 metabolites as monitored by UHPLC from larvae in which Spen was, in a FB-specific manner, either depleted via RNAi or overexpressed, as compared to controls (FB w-RNAi or GFP overexpression controls, respectively). For each of three biological replicates, ten individual larvae were analyzed per genotype. *n* = 30.
(XLSX)

S2 Data As measured by RNA sequencing, levels of transcripts that changed significantly between FB spen-RNAi and overexpression when compared to appropriate controls (FB w-RNAi or GFP overexpression controls, respectively). For each of three biological replicates, forty dissected FBs were pooled together and analyzed per genotype. *n* = 3.
(ZIP)
