# Correction: An autonomous metabolic role for Spen

**DOI:** 10.1371/journal.pgen.1007266

**Published:** 2018-03-06

**Authors:** Kelsey E. Hazegh, Travis Nemkov, Angelo D’Alessandro, John D. Diller, Jenifer Monks, James L. McManaman, Kenneth L. Jones, Kirk C. Hansen, Tânia Reis

In S1 Fig, panels B and C have been labelled incorrectly. Panel B should be labelled “Male” and panel C should be labelled “Female”. Please view the correct S1 Fig below.

In the eighth sentence under the subheading “Spen is necessary and sufficient to reduce fat accumulation in the fat body” in the Results section, the word “males” should read “females” and vice versa. The correct sentence is: Notably, although males of all genotypes stored more fat than females, for both sexes the increase in buoyancy resulting from Spen depletion was similar (mean fold change for all sucrose concentrations ± SEM, 7.9 ± 1.5 for males and 6.0 ± 1.3 for females, P = 0.34 by unpaired *t* test) (S1B and S1C Fig).

## Supporting information

S1 Fig Knockdown of Spen results in a low-density phenotype. (A) Percent of floating larvae in different density solutions. FB-specific Spen KD (dcg>iSpen, BL33398) as in Fig 1A with additional dcg/+ background control. Fifty larvae per genotype per experimental replicate, *n* = 8 biological replicates per genotype. (B) Percent of male only Spen KD larvae floating. (C) Percent of female only Spen KD larvae floating. (D) FB-specific Spen KD (dcg>iSpen, BL50529) with different insertion site as Spen KD in Fig 1A compared to KD control (dcg>iw). (E) Genetic background controls (iSpen/+ and iw/+) for (D). (F) As the Spen hairpin insertion site appears to result in a lean phenotype, KD animals were normalized to their genetic background. (G) As in (A), three additional independent Spen hairpin constructs (dcg>iSpen) tested in different density solutions and compared to KD control (dcg>iw). (H) Genetic background controls (iSpen/+’s and iw/+) for (G). P value obtained by ANOVA. *P < 0.05, ** P < 0.01, ***P < 0.001, **** P < 0.0001. Error bars represent SEM.
(TIF)
