# Supplementary figures and images for: An autonomous metabolic role for Spen
Source: PLoS Genet. 2017 Jun 22;13(6):e1006859. doi: 10.1371/journal.pgen.1006859 (PMC5501677; doi:10.1371/journal.pgen.1006859)

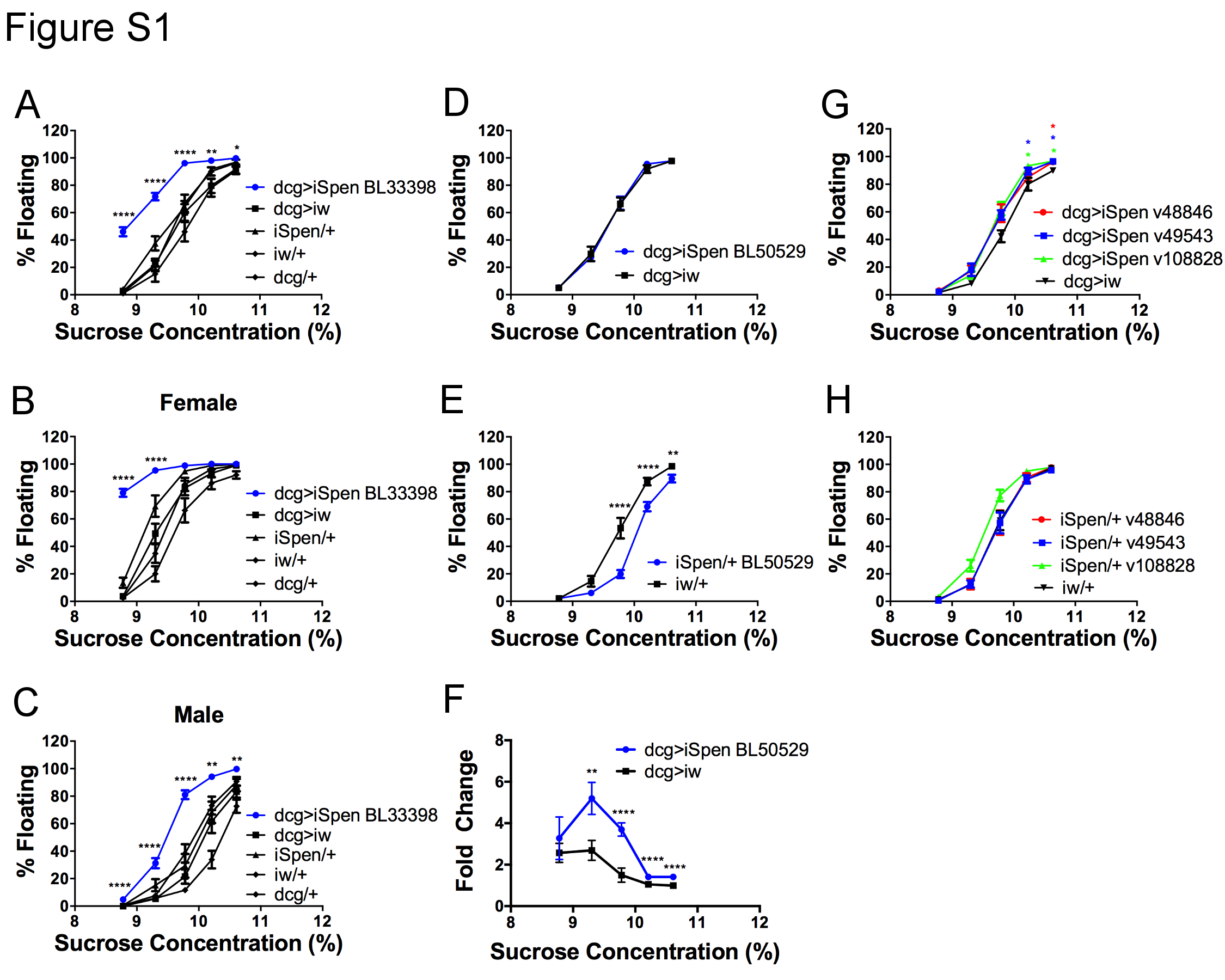

Supplement: S1 Fig — (A)Percent of floating larvae in different density solutions. FB-specific Spen KD (dcg>iSpen, BL33398) as in Fig 1A with additional dcg/+ background control. Fifty larvae per genotype per experimental replicate, n = 8 biological replicates per genotype.(B)Percent of female only Spen KD larvae floating.(C)Percent of male only Spen KD larvae floating.(D)FB-specific Spen KD (dcg>iSpen, BL50529) with different insertion site as Spen KD in Fig 1A compared to KD control (dcg>iw).(E)Genetic background controls (iSpen/+ and iw/+) for (D).(F)As the Spen hairpin insertion site appears to result in a lean phenotype, KD animals were normalized to their genetic background.(G)As in (A), three additional independent Spen hairpin constructs (dcg>iSpen) tested in different density solutions and compared to KD control (dcg>iw).(H)Genetic background controls (iSpen/+’s and iw/+) for (G). P value obtained by ANOVA. *P < 0.05, ** P < 0.01, ***P < 0.001, **** P < 0.0001. Error bars represent SEM. (TIF) [file pgen.1006859.s001.tif]

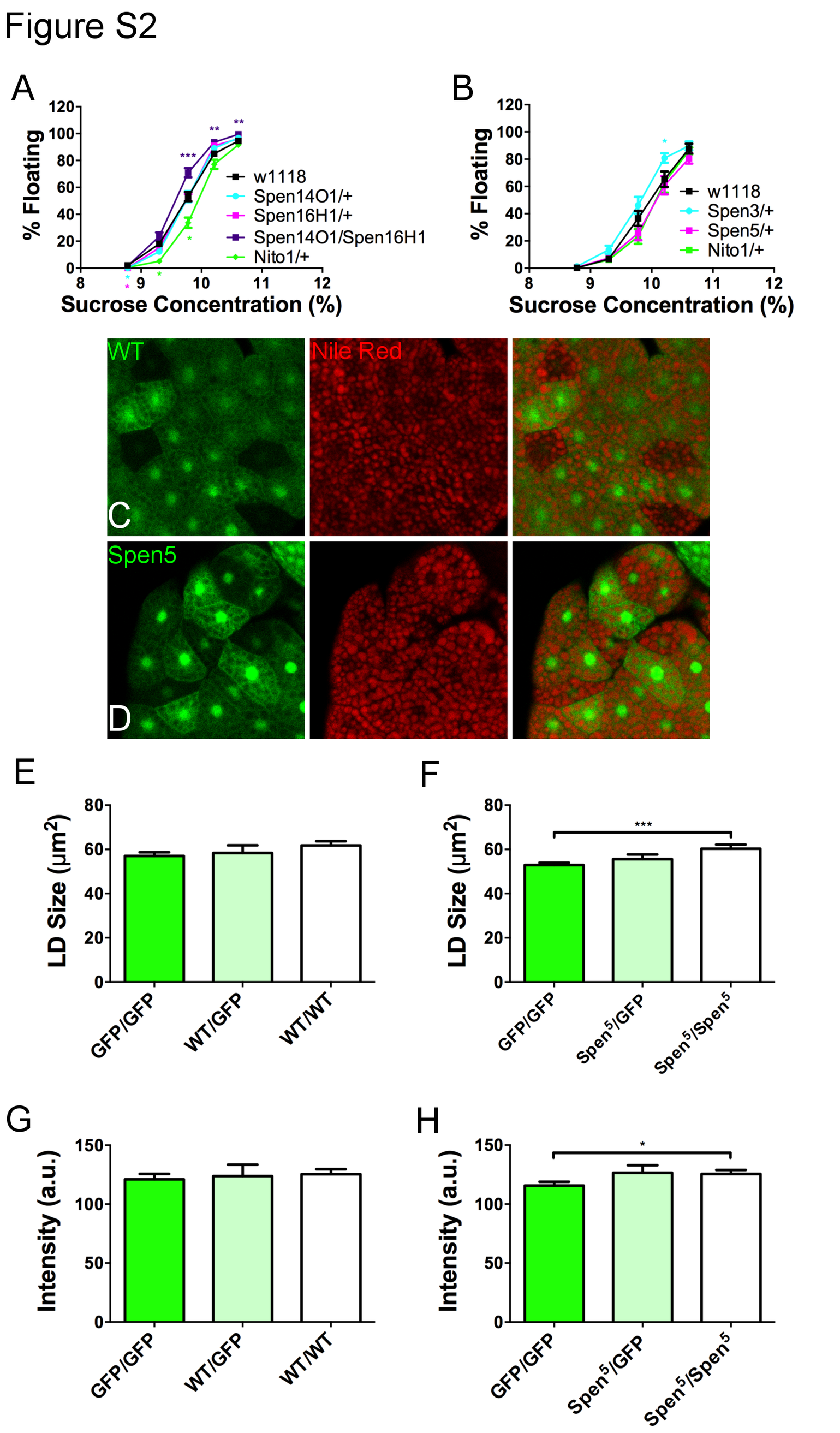

Supplement: S2 Fig — (A)Percent of floating larvae in different density solutions. Heterozygous Spen and Nito mutants compared to w1118 control. w1118 control and mutants were backcrossed to w; sco/cyo GFP for two generations. Fifty larvae per genotype per experimental replicate, n = 8–16 biological replicates per genotype. Error bars represent SEM.(B)As in (A), additional heterozygous Spen null mutants and Nito mutant compared to w1118 control. w1118 control and mutants were backcrossed to w; sco/cyo GFP for six generations.(C-D) Larval FB tissue from heterozygous Spen5 mutant animals expressing WT (bright green) or fully mutant (no green) clonally. Tissues stained with the lipophilic dye Nile Red to mark neutral lipids (red). (C) WT control, (D) Spen5. Error bars represent SEM.(E-F) Lipid droplet (LD) size in WT control (E) or Spen5 (F) clones. GFP/GFP represents fully WT cells, GFP/WT or Spen5 represents heterozygous cells, and WT/WT or Spen5/Spen5 represents recombined mutant (or control) cells. (E) GFP/GFP n = 143, WT/WT n = 191, (F) GFP/GFP n = 175, Spen5/Spen5 n = 184. Error bars represent SEM. P value obtained by unpaired two-tailed t tests.(G-H) LD intensity in WT control (G) or Spen5 (H) clones. (E) GFP/GFP n = 143, WT/WT n = 191, (F) GFP/GFP n = 175, Spen5/Spen5 n = 184. Error bars represent SEM. P value obtained by unpaired two-tailed t tests. *P < 0.05, ** P < 0.01, ***P < 0.001, **** P < 0.0001. (TIF) [file pgen.1006859.s002.tif]

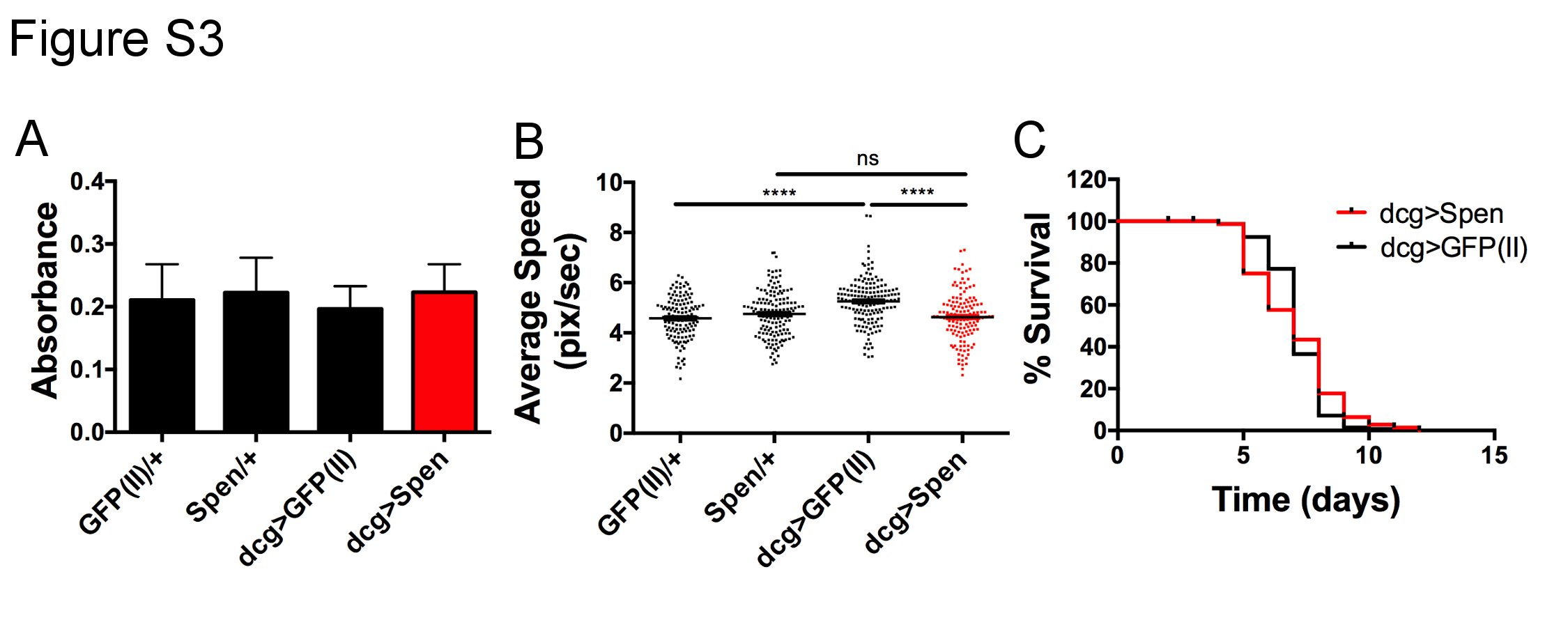

Supplement: S3 Fig — (A)Absorbance at 530 nm as a measure of food intake for Spen overexpression (dcg>Spen) compared to overexpression control (dcg>GFP) and genetic background controls (Spen/+ and GFP/+), n = 4. P value obtained by ANOVA. Error bars represent SD.(B)Average larval speed, pixels/sec. n = 4. P value obtained by unpaired two-tailed t test. Error bars represent SEM.(C)Larvae reared in amino acid-free media and tracked for survival. Fifty larvae per genotype per experimental replicate, n = 3. P value obtained by Log-rank test. *P < 0.05, ** P < 0.01, ***P < 0.001, **** P < 0.0001. (TIF) [file pgen.1006859.s003.tif]

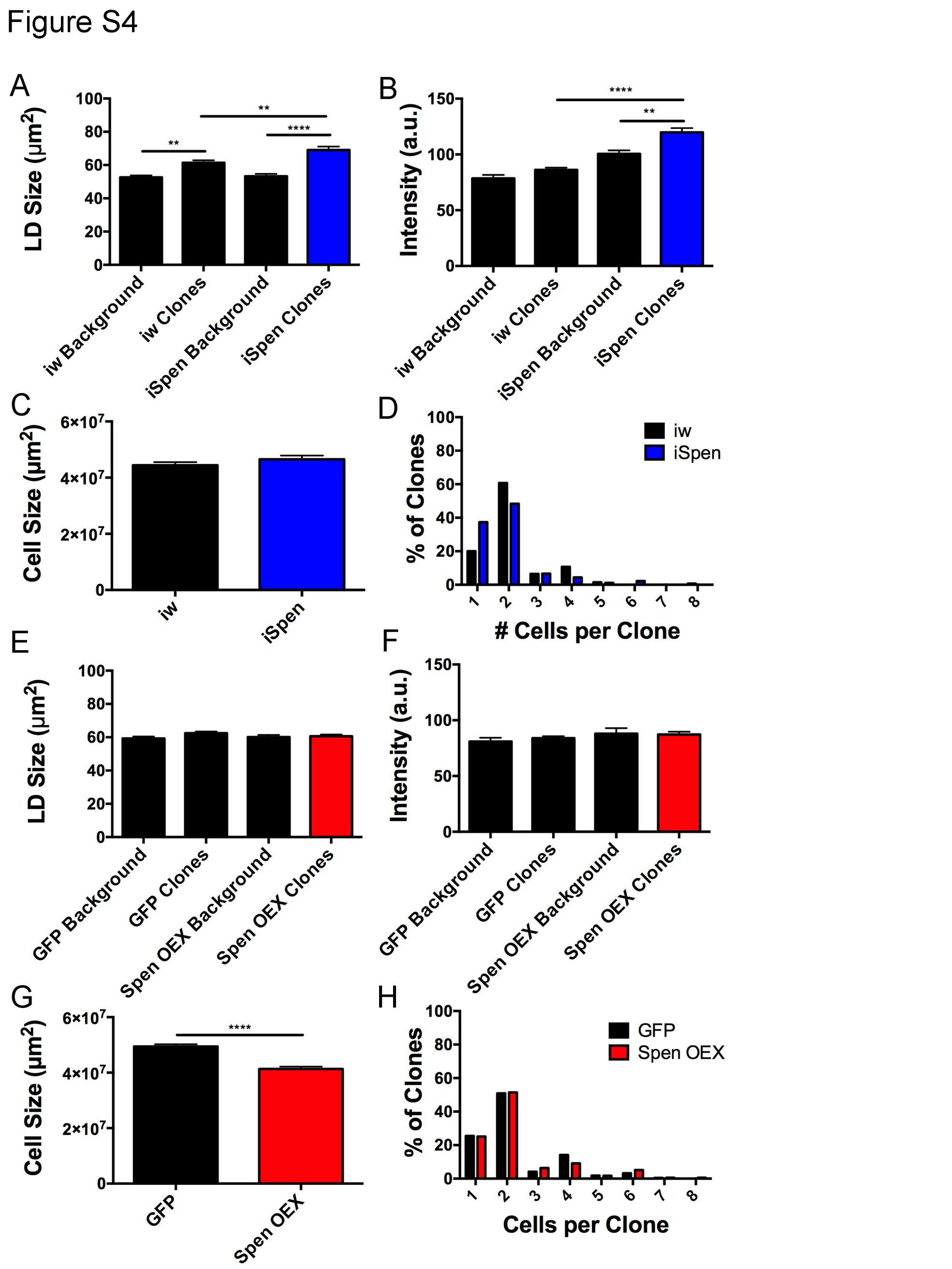

Supplement: S4 Fig — (A)Lipid droplet (LD) size in Spen KD (iSpen) clones or KD control (iw) clones compared to non-clone cells (denoted as background). Spen KD n = 91. W KD n = 140.(B)LD intensity in Spen KD or KD control clones compared to non-clone cells.(C)FB cell size of Spen KD and KD control clones.(D)Percentage of numbers of cells within each clone of Spen KD compared to KD control. P value obtained by ANOVA.(E)As in (A), LD size in Spen OEX clones compared to OEX control (GFP) clones and non-clone cells (denoted as background). Spen OEX n = 175. GFP n = 220.(F)As in (B), LD intensity in Spen OEX clones compared to OEX control clones and non-clone cells.(G)As in (C), FB cell size of Spen OEX clones compared to OEX control clones.(H)As in (D), percentage of number of cells per clone of Spen OEX compared to OEX control. Error bars represent SEM. P values obtained by unpaired two-tailed t test. * P < 0.05, ** P < 0.01, ***P < 0.001, **** P < 0.0001. (TIF) [file pgen.1006859.s004.tif]

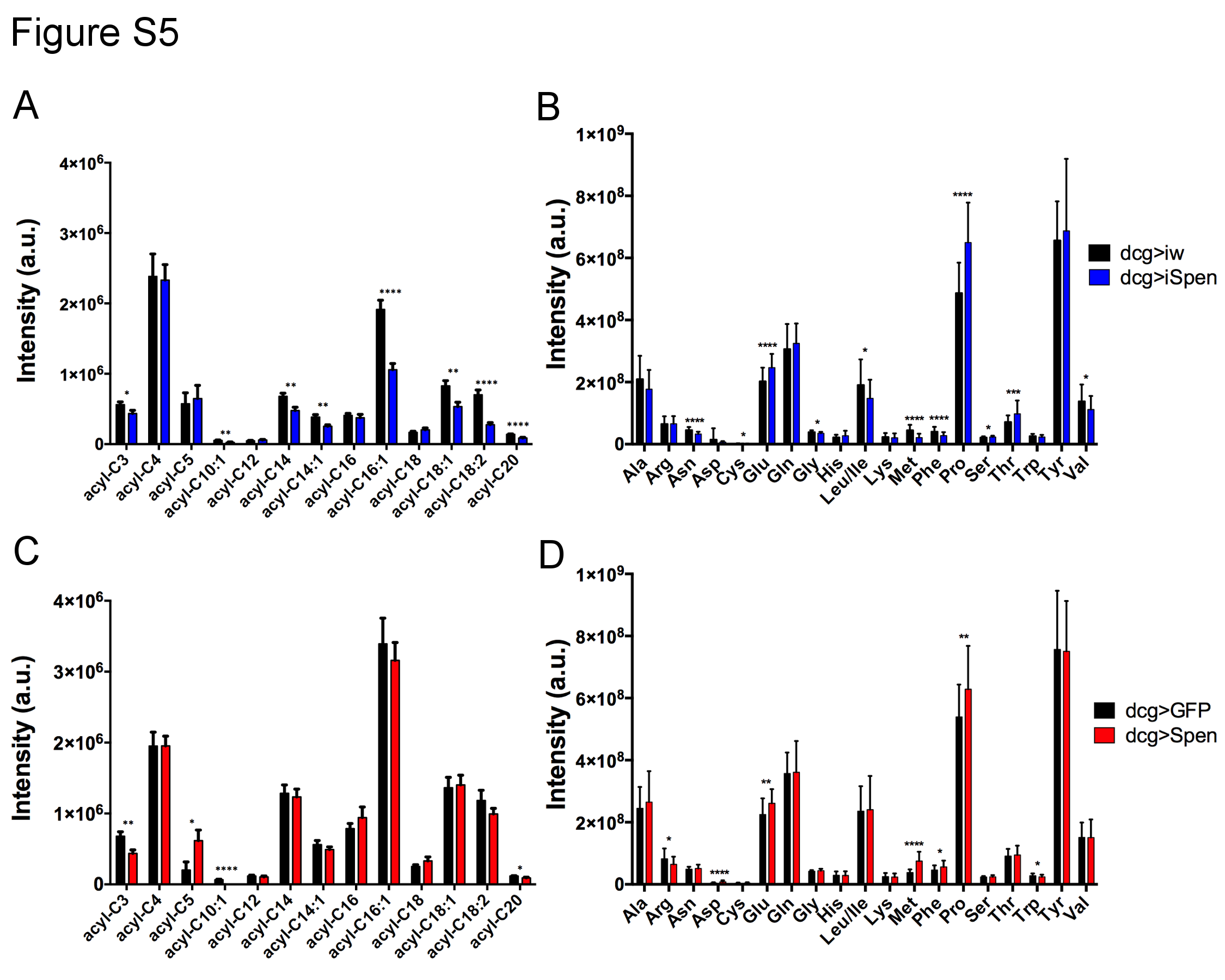

Supplement: S5 Fig — (A)Levels of acyl-carnitines in Spen KD larvae (dcg>iSpen) compared to KD control (dcg>iw) as determined by UHPLC.(B)Levels of amino acids in Spen KD larvae compared to w KD as determined by UHPLC.(C)As in (A), Spen OEX (dcg>Spen) compared to OEX control (dcg>GFP).(D)As in (B), Spen OEX compared to OEX control. P values obtained by unpaired two-tailed t test. * P < 0.05, ** P < 0.01, ***P < 0.001, **** P < 0.0001. Error bars represent SD. (TIF) [file pgen.1006859.s005.tif]

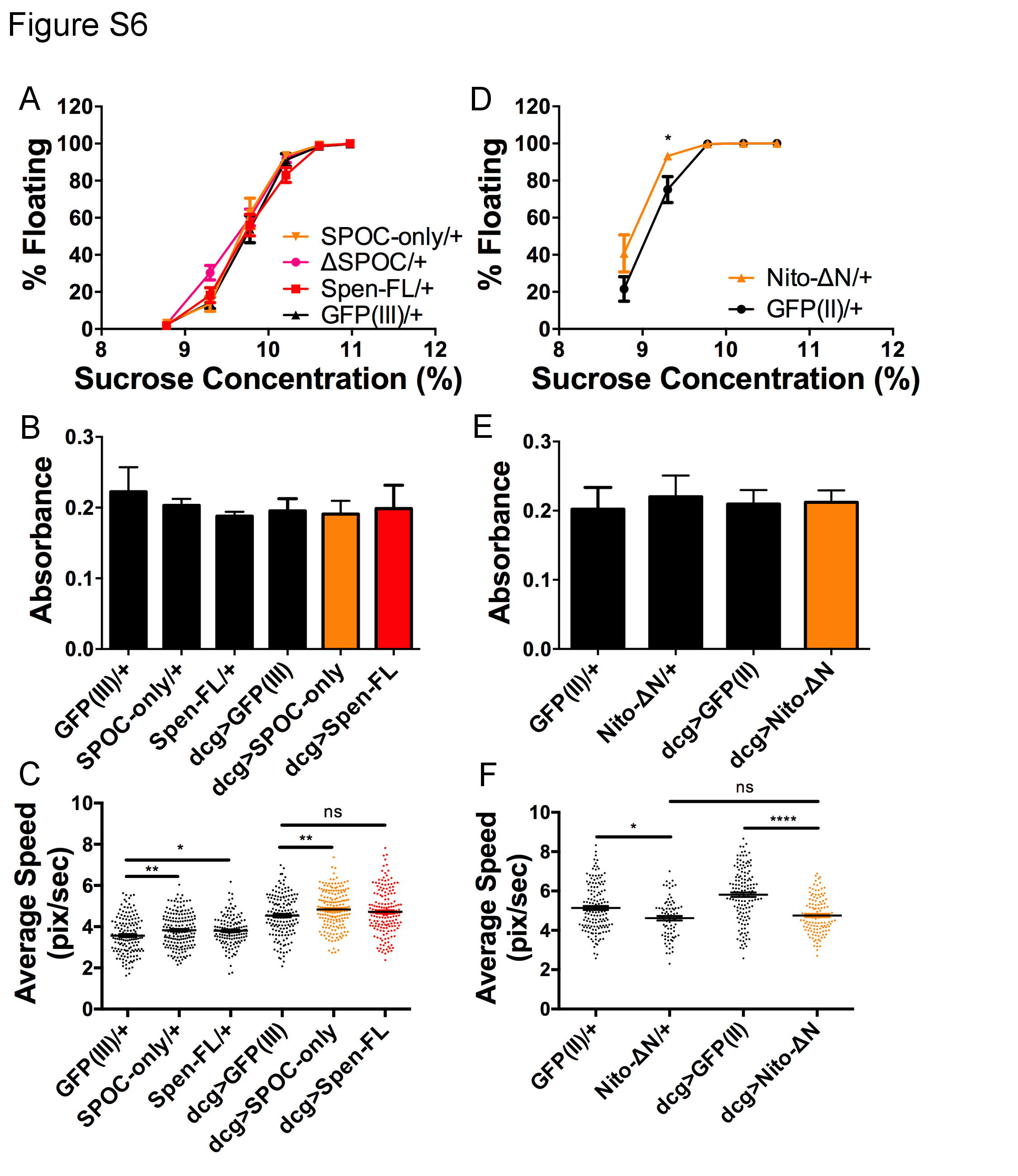

Supplement: S6 Fig — (A)Percent of floating larvae in different density solutions. Genetic background controls for Fig 3F. Fifty larvae per genotype per experimental replicate, n = 8 biological replicates per genotype. P values obtained by ANOVA. Error bars represent SEM.(B and E) Absorbance at 530 nm as a measure of food intake, n = 4. (B) FB specific OEX of Spen-FL (dcg>Spen-FL) and SPOConly (dcg>SPOC-only) compared to OEX control (dcg>GFP) and genetic background controls (Spen-FL/+, SPOC-only/+, and GFP/+), (E) FB-specific OEX of Nito-ΔN (dcg>Nito-ΔN) compared to OEX control (dcg>GFP) and genetic background controls (Nito-ΔN /+ and GFP/+). P values obtained by ANOVA. Error bars represent SD.(C and F) Average larval speed, pixels/sec. n = 4. P values obtained by unpaired two-tailed t test. Error bars represent SEM.(D)As in (A), genetic background controls for Fig 3G. *P < 0.05, ** P < 0.01, ***P < 0.001, **** P < 0.0001. (TIF) [file pgen.1006859.s006.tif]

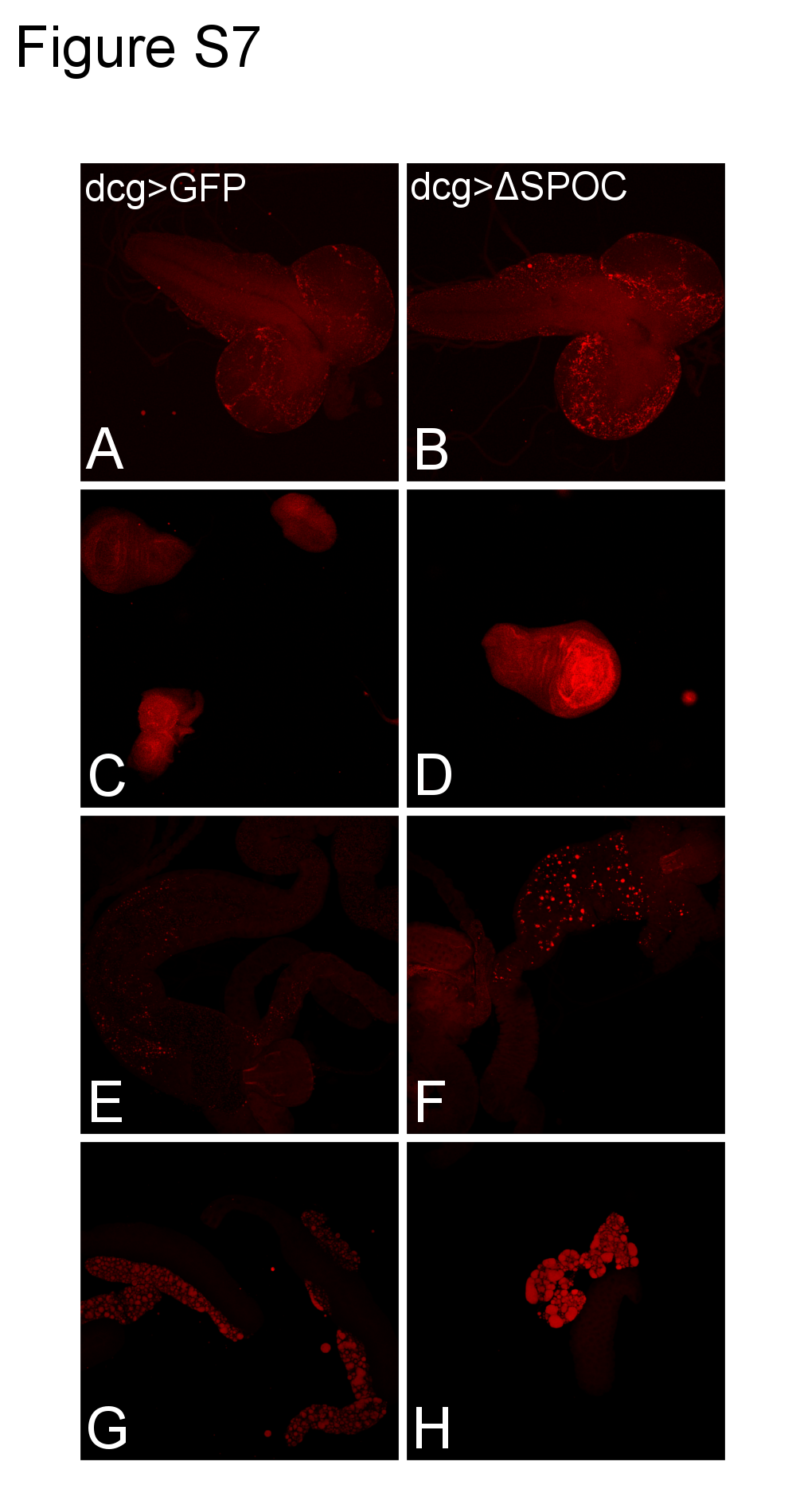

Supplement: S7 Fig — (A and B) Larvae reared at 16°C and collected at wandering stage. (A) dcg>GFP and (B) dcg>ΔSPOC larval brains stained with Nile Red.(C and D) Imaginal discs.(E and F) Guts.(G and H) Salivary Glands. (TIF) [file pgen.1006859.s007.tif]

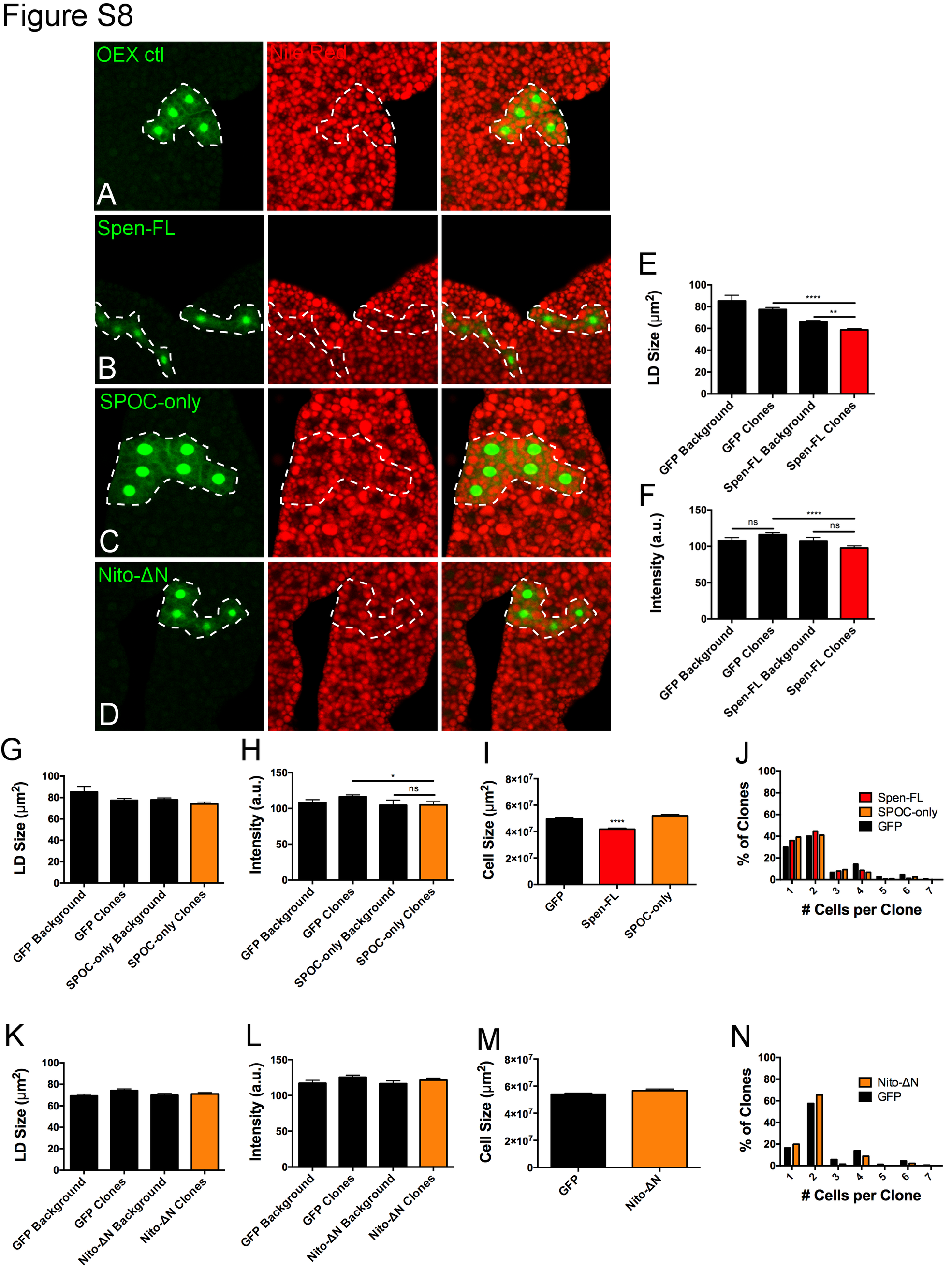

Supplement: S8 Fig — (A-D) Larval FB tissue ectopically expressing constructs along with GFP (green). Tissues stained with the lipophilic dye Nile Red to mark neutral lipids (red). Dotted white line outlines construct-expressing clones. (A) UAS-GFP (as in Fig 4J), (B) UAS-Spen-FL, (C) UAS-Spen-SPOConly, (D) UAS-Nito-ΔN. Clones were obtained without heat shock induction of flp but instead from “leaky” flp expression during FB development.(E)Lipid droplet (LD) size in Spen-FL or OEX control (GFP) clones compared to non-clone cells (denoted as background). Spen-FL n = 161. GFP n = 147.(F)LD intensity in Spen-FL or control clones compared to non-clone cells.(G)As in (E), LD size in Spen-SPOConly clones compared to control clones and non-clone cells. Spen-SPOConly n = 117. GFP n = 147.(H)As in (F), LD intensity in Spen-SPOConly clones compared to control clones and non-clone cells.(I)FB cell size of Spen-FL, Spen-SPOConly, and control clones.(J)Percentage of numbers of cells within each clone of Spen-FL and Spen-SPOConly compared to control. P value obtained by ANOVA.(K)As in (E), LD size in Nito-ΔN clones compared to control clones and non-clone cells. Nito-ΔN n = 136. GFP n = 158.(L)As in (F), LD intensity in Nito-ΔN clones compared to control clones and non-clone cells.(M)As in (I), FB cell size of Nito-ΔN clones and control clones.(N)As in (J), percentage of number of cells within each clone of Nito-ΔN compared to control. Error bars represent SEM. P values obtained by unpaired two-tailed t test. * P < 0.05, ** P < 0.01, ***P < 0.001, **** P < 0.0001. (TIF) [file pgen.1006859.s008.tif]

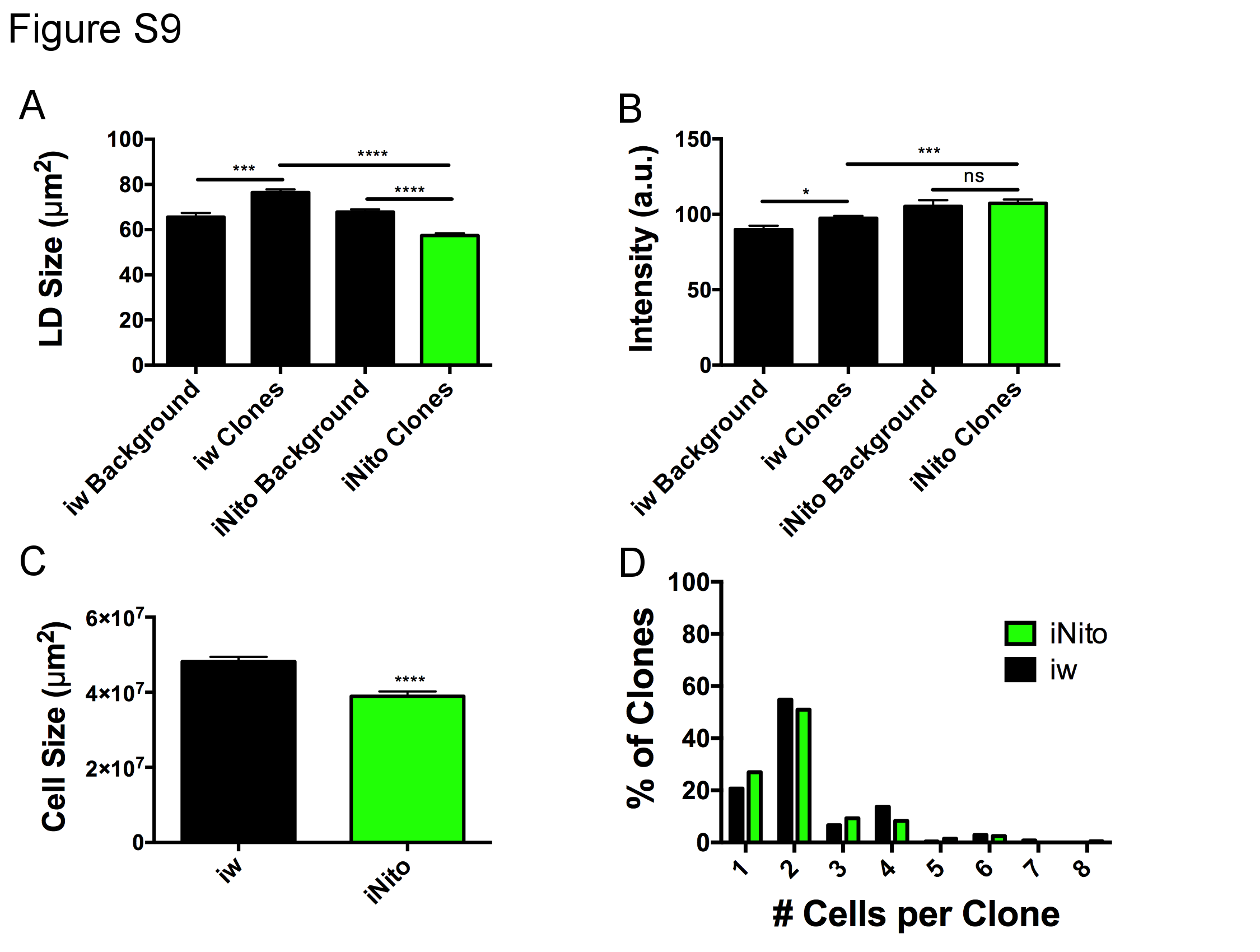

Supplement: S9 Fig — (A)Lipid droplet (LD) size in Nito KD (iNito) or control (iw) clones compared to non-clone cells (denoted as background). Nito KD n = 204. w KD n = 241.(B)LD intensity in Nito KD or control clones compared to non-clone cells.(C)FB cell size of Nito KD and control clones.(D)Percentage of numbers of cells within each clone of Nito KD compared to control. P value obtained by ANOVA. Error bars represent SEM. P values obtained by unpaired two-tailed t test. *P < 0.05, ** P < 0.01, ***P < 0.001, **** P < 0.0001. (TIF) [file pgen.1006859.s009.tif]

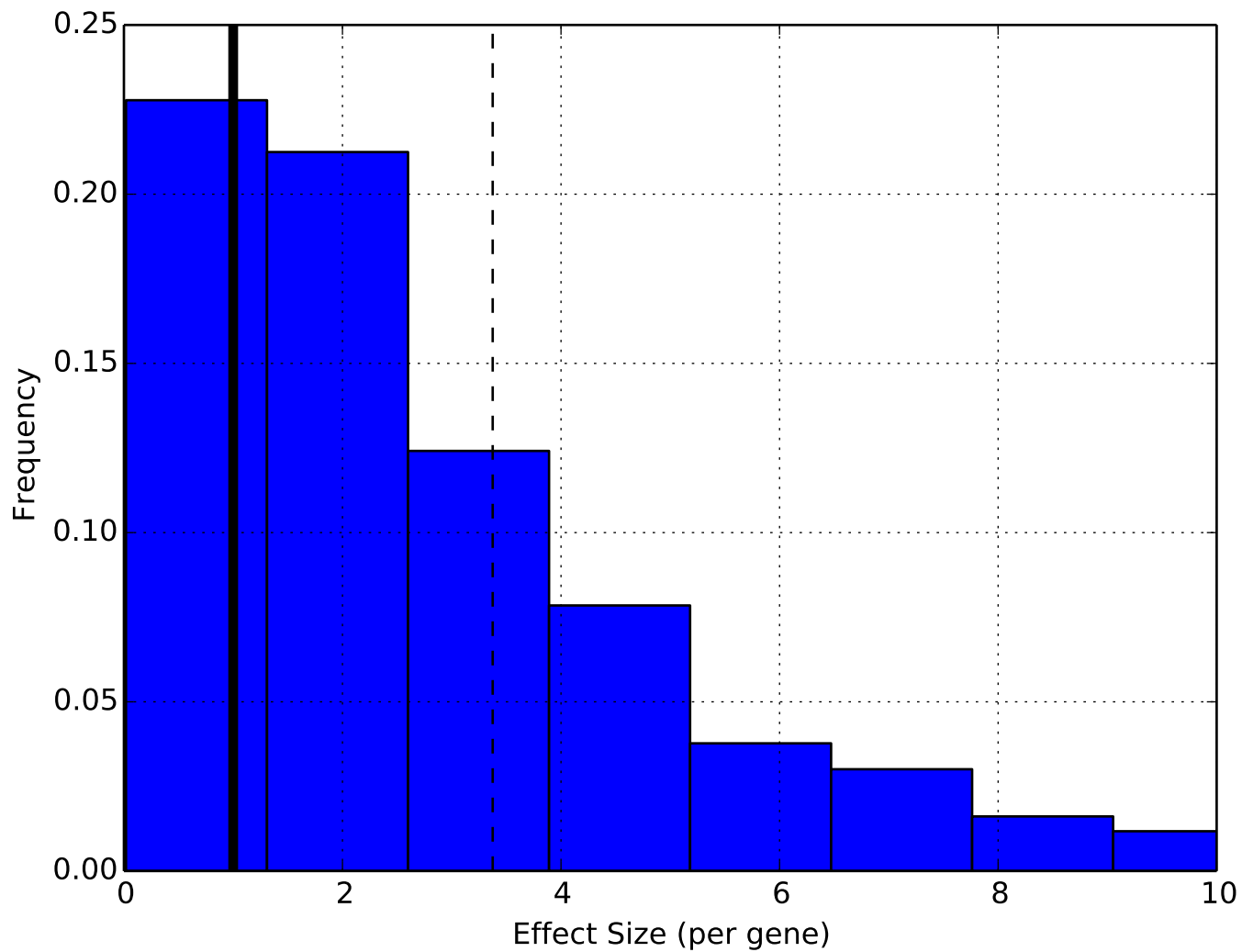

Supplement: S2 Data — For each of three biological replicates, forty dissected FBs were pooled together and analyzed per genotype. n = 3. (ZIP) [file pgen.1006859.s013.zip › Reis_results/Reis_Ex1_effect_size_histogram.pdf]

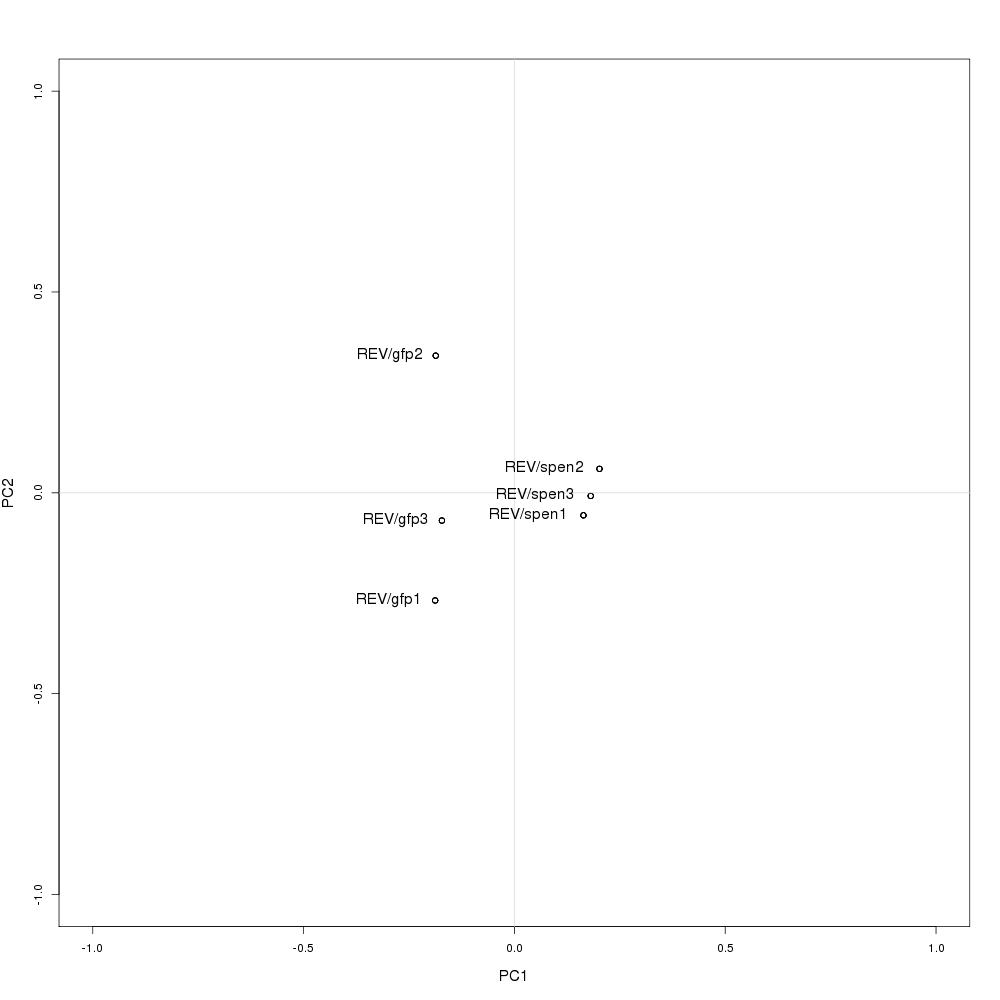

Supplement: S2 Data — For each of three biological replicates, forty dissected FBs were pooled together and analyzed per genotype. n = 3. (ZIP) [file pgen.1006859.s013.zip › Reis_results/Reis_Ex1_PCA_plot.png]

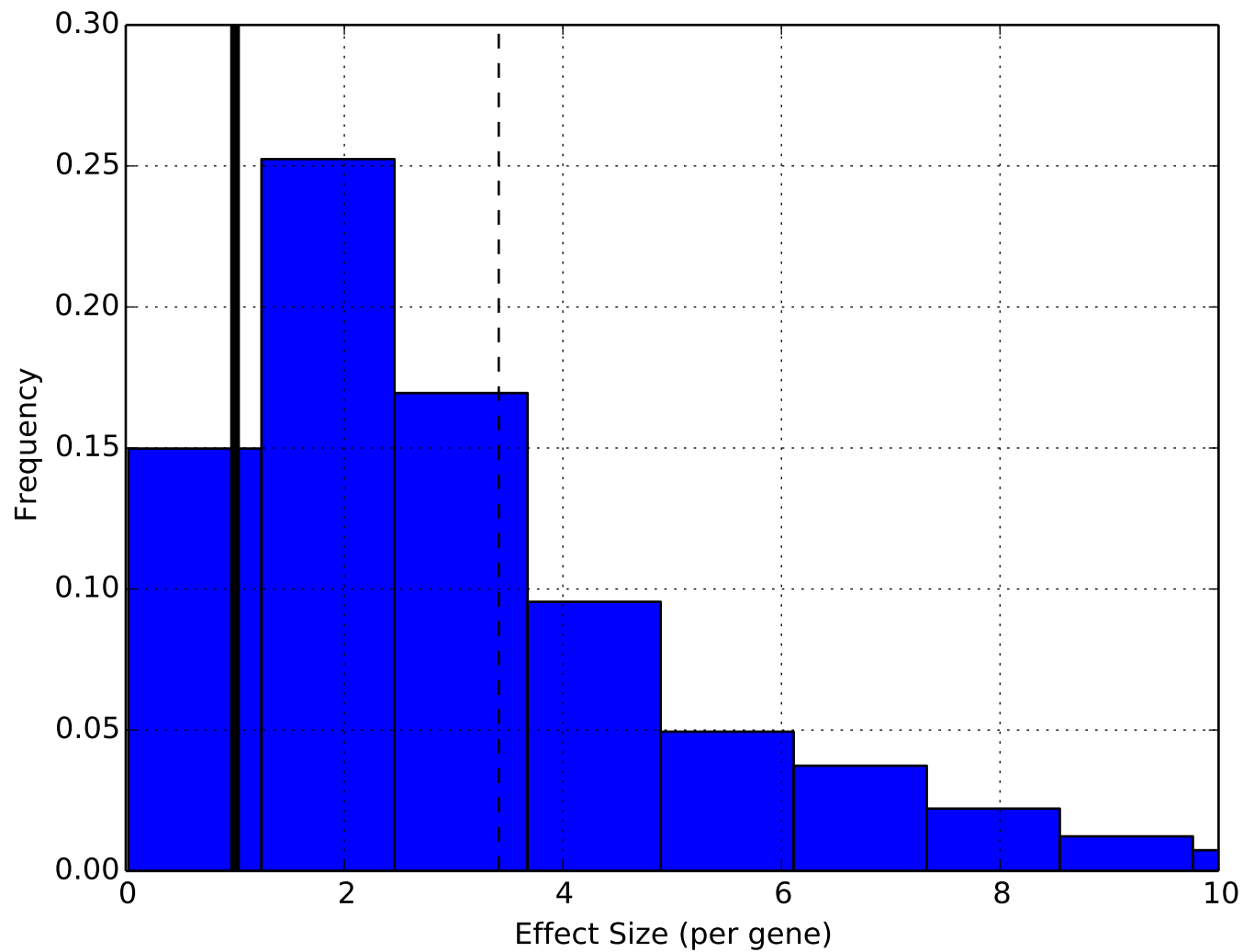

Supplement: S2 Data — For each of three biological replicates, forty dissected FBs were pooled together and analyzed per genotype. n = 3. (ZIP) [file pgen.1006859.s013.zip › Reis_results/Reis_Ex2_effect_size_histogram.pdf]

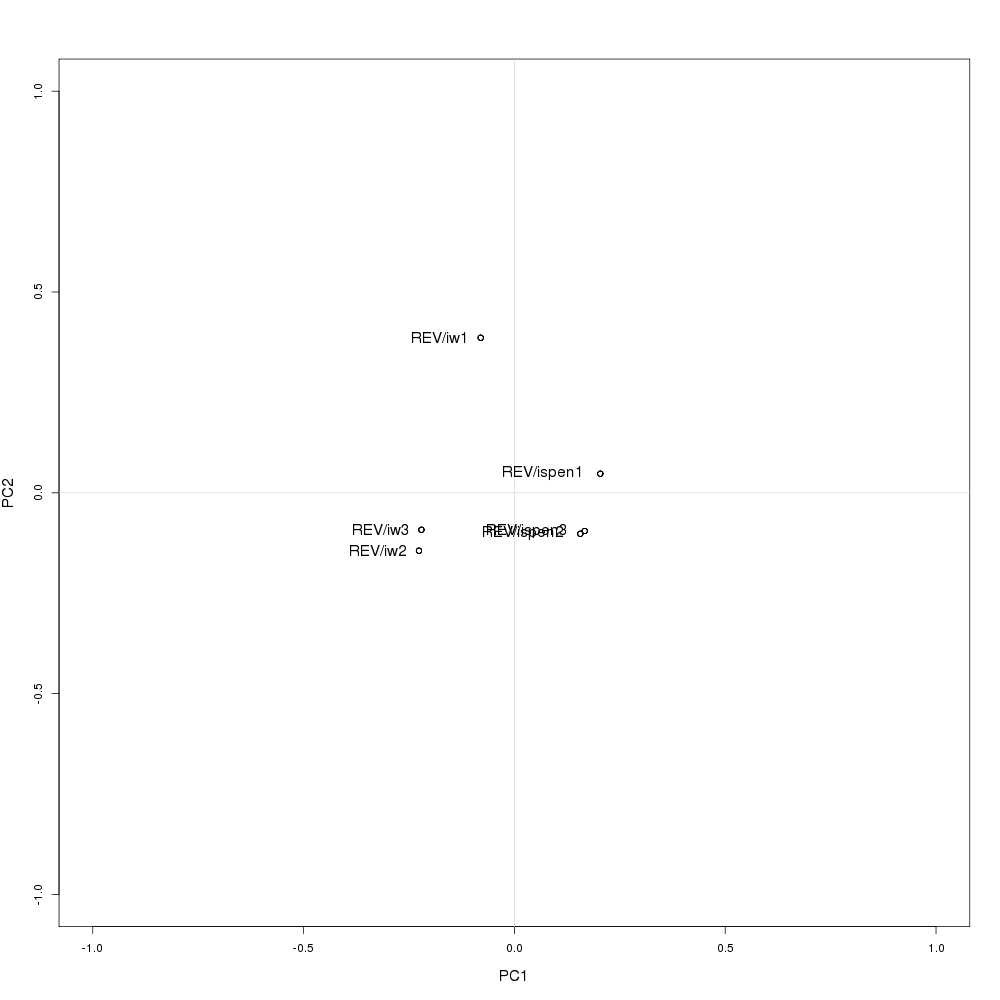

Supplement: S2 Data — For each of three biological replicates, forty dissected FBs were pooled together and analyzed per genotype. n = 3. (ZIP) [file pgen.1006859.s013.zip › Reis_results/Reis_Ex2_PCA_plot.png]

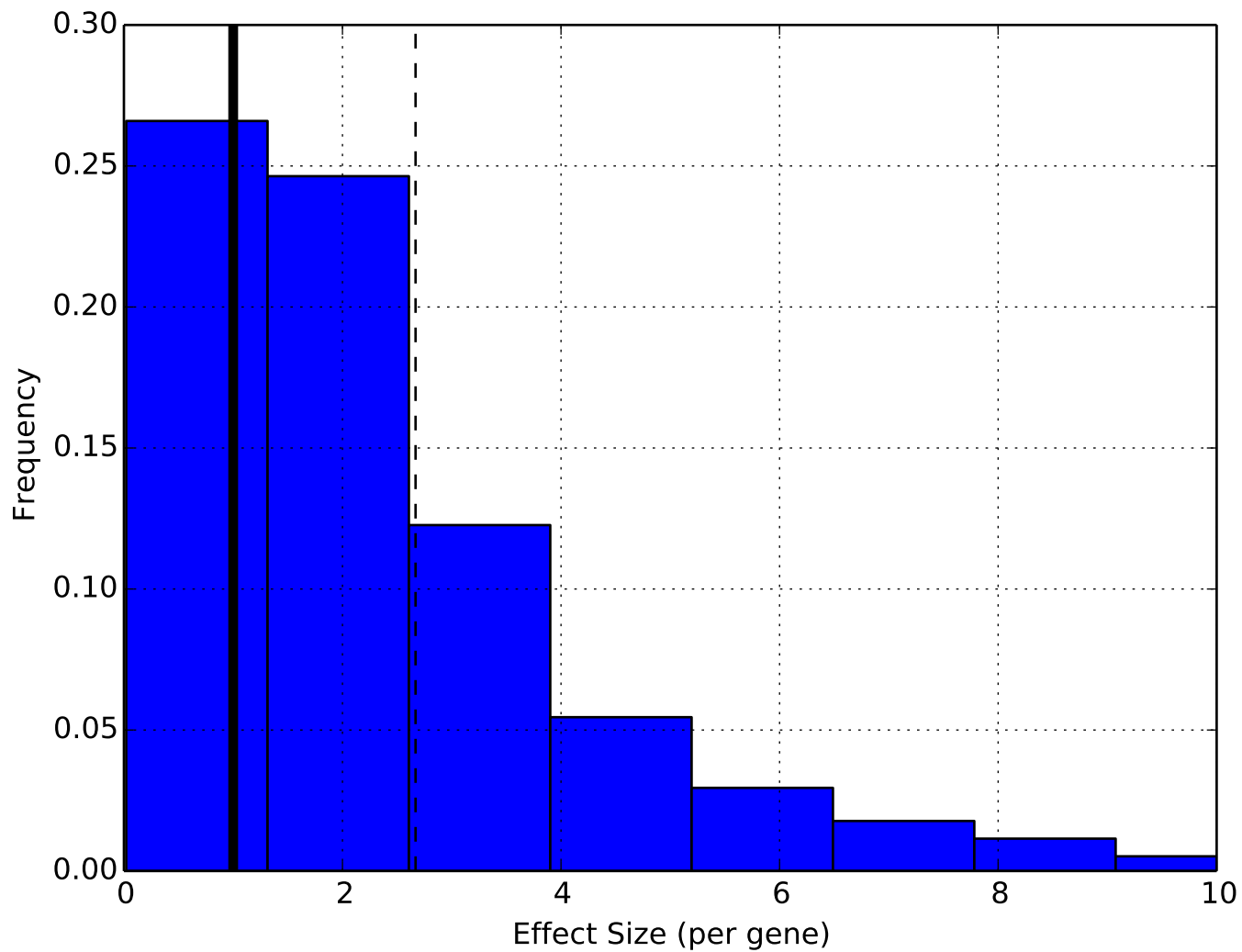

Supplement: S2 Data — For each of three biological replicates, forty dissected FBs were pooled together and analyzed per genotype. n = 3. (ZIP) [file pgen.1006859.s013.zip › Reis_results/Reis_Ex3_effect_size_histogram.pdf]

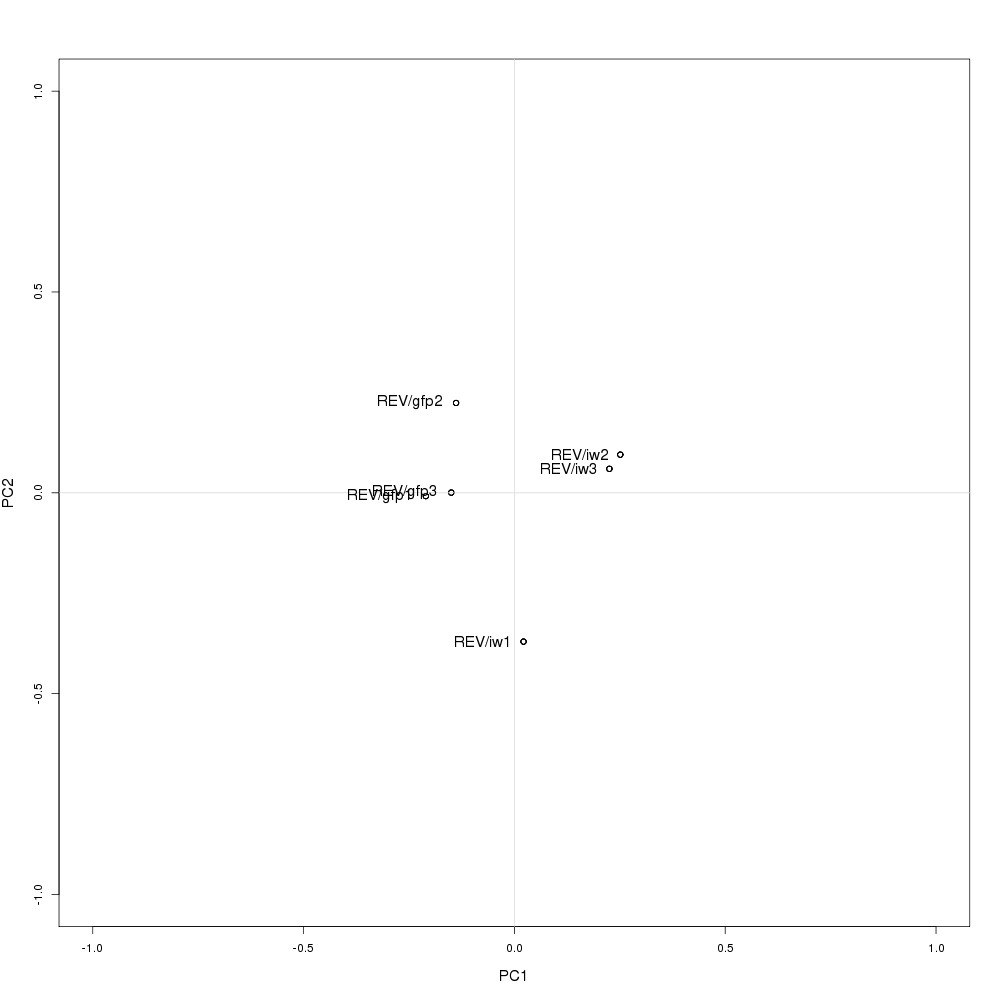

Supplement: S2 Data — For each of three biological replicates, forty dissected FBs were pooled together and analyzed per genotype. n = 3. (ZIP) [file pgen.1006859.s013.zip › Reis_results/Reis_Ex3_PCA_plot.png]

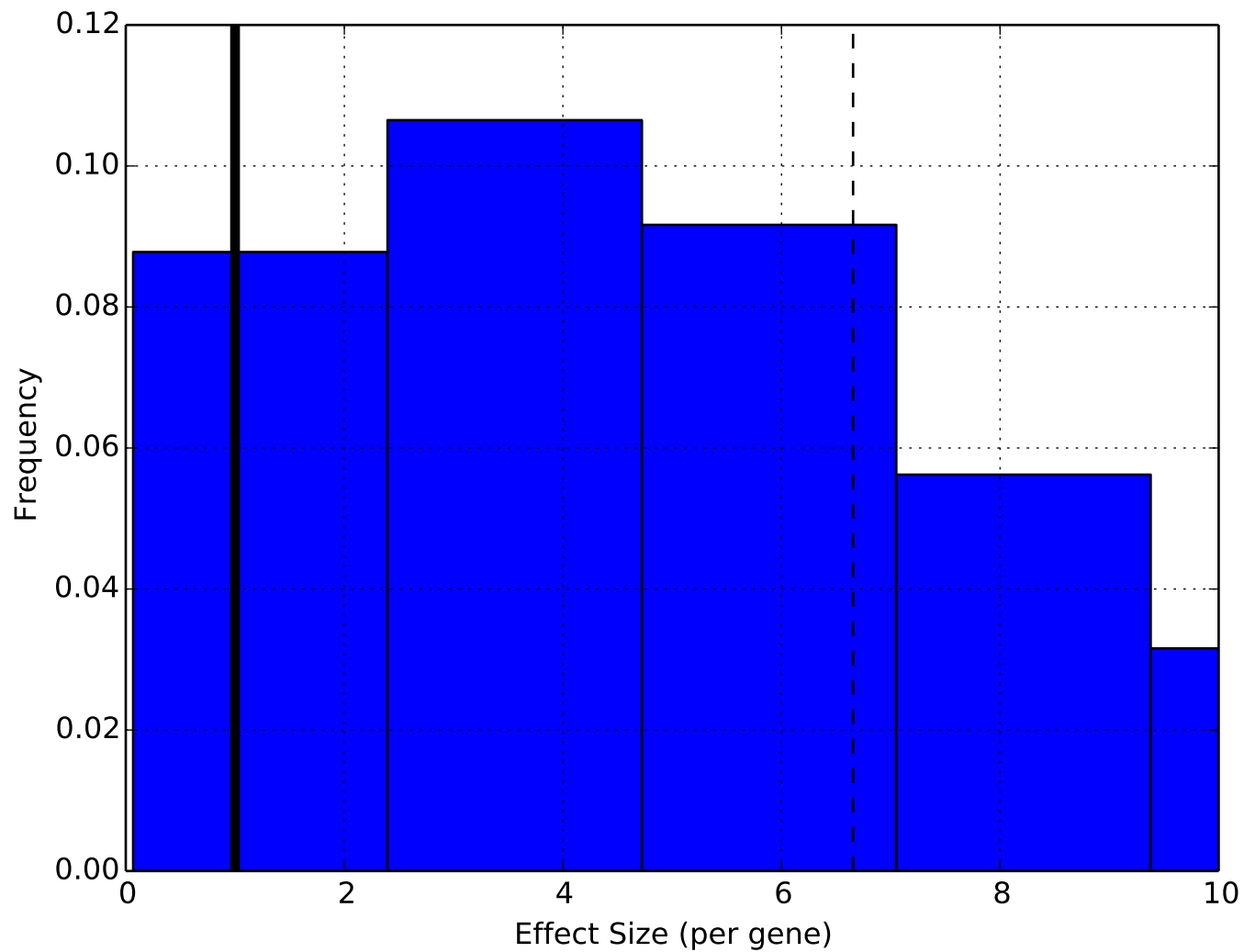

Supplement: S2 Data — For each of three biological replicates, forty dissected FBs were pooled together and analyzed per genotype. n = 3. (ZIP) [file pgen.1006859.s013.zip › Reis_results/Reis_Ex4_effect_size_histogram.pdf]

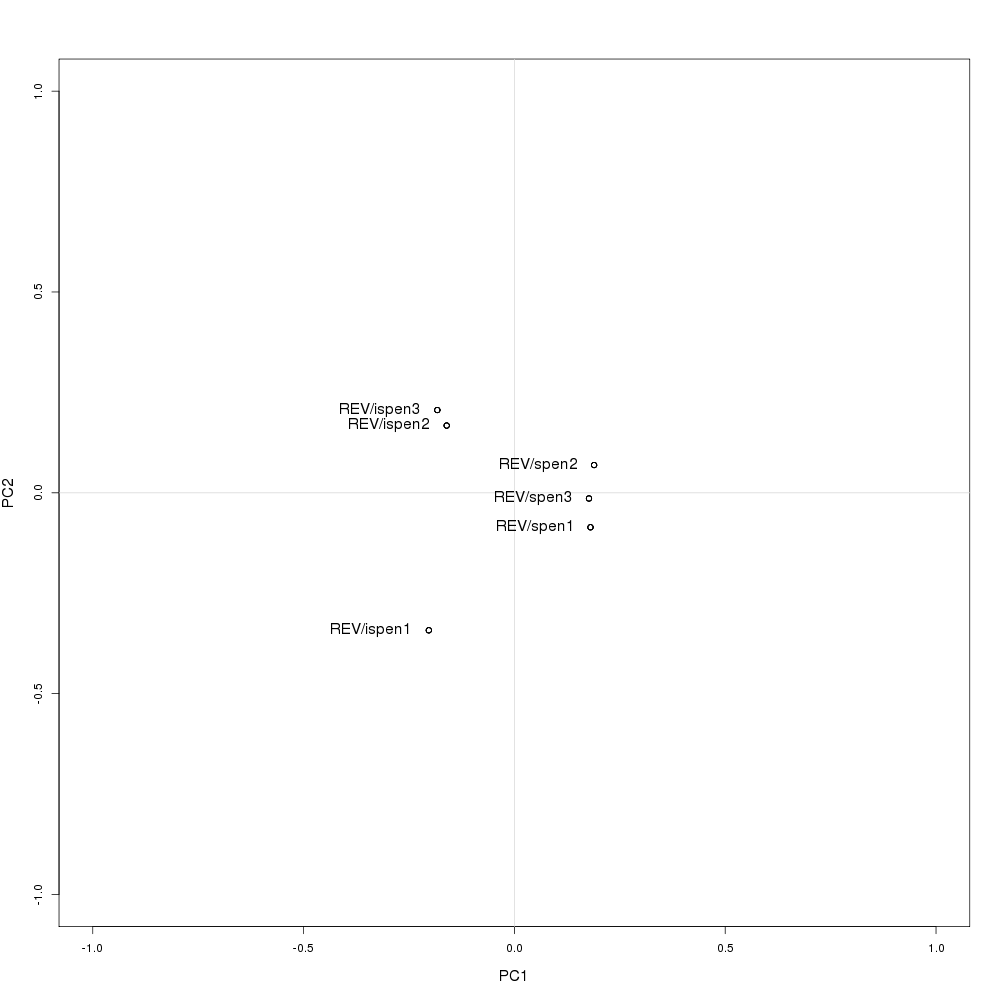

Supplement: S2 Data — For each of three biological replicates, forty dissected FBs were pooled together and analyzed per genotype. n = 3. (ZIP) [file pgen.1006859.s013.zip › Reis_results/Reis_Ex4_PCA_plot.png]
