# Supplementary material for: An autonomous metabolic role for Spen
Source: PLoS Genet. 2017 Jun 22;13(6):e1006859. doi: 10.1371/journal.pgen.1006859 (PMC5501677; doi:10.1371/journal.pgen.1006859)
Supplement: S1 Table — (DOCX) [file pgen.1006859.s010.docx]

| Genes Upregulated Upon Both Spen KD and Starvation | | | |
| --- | --- | --- | --- |
| Gene | **FlyBase ID** | **Gene** | **FlyBase ID** |
| CG10799 | FBgn0033821 | **wtrw** | FBgn0260005 |
| Cyp4p1 | FBgn0015037 | **CG32557** | FBgn0052557 |
| CG10924 | FBgn0034356 | **Sp212** | FBgn0053329 |
| CG11529 | FBgn0036264 | **CG4025** | FBgn0025624 |
| CG13704 | FBgn0035583 | **pug** | FBgn0020385 |
| PGRP-SC2 | FBgn0043575 | **Idgf1** | FBgn0020416 |
| CG15065 | FBgn0040734 | **eIF3-S9** | FBgn0034237 |
| CG15067 | FBgn0034331 | **loco** | FBgn0020278 |
| CG15068 | FBgn0040733 | **sra** | FBgn0086370 |
| CG16743 | FBgn0032322 | **cert** | FBgn0027569 |
| IM3 | FBgn0040736 | **puc** | FBgn0243512 |
| AdSS | FBgn0027493 | **Strica** | FBgn0033051 |
| Pepck | FBgn0003067 | **CG8160** | FBgn0034011 |
| CG18107 | FBgn0034330 | **CG8299** | FBgn0034052 |
| IMPPP | FBgn0283462 | **CG8788** | FBgn0028955 |
| CG18473 | FBgn0037683 | **Non1** | FBgn0028473 |
| CG1882 | FBgn0033226 | **Cyp28a5** | FBgn0028940 |
| CG2017 | FBgn0037391 | **Tsp29Fb** | FBgn0032075 |
| Fer1HCH | FBgn0015222 | **CR10102** | FBgn0033927 |
| Obp49a | FBgn0050052 |  |  |

**Table S1:** Genes that are upregulated upon both Spen depletion in the FB and under starvation conditions [63].
