# Supplementary material for: An autonomous metabolic role for Spen
Source: PLoS Genet. 2017 Jun 22;13(6):e1006859. doi: 10.1371/journal.pgen.1006859 (PMC5501677; doi:10.1371/journal.pgen.1006859)
Supplement: S2 Table — (DOCX) [file pgen.1006859.s011.docx]

| Genes Downregulated Upon Both Spen KD and Starvation | | | |  |
| --- | --- | --- | --- | --- |
| Gene | | **FlyBase ID** | **Gene** | **FlyBase ID** |
| CG10674 | | FBgn0035592 | **Dhpr** | FBgn0035964 |
| Nedd8 | | FBgn0032725 | **CG4729** | FBgn0036623 |
| CG1092 | | FBgn0037228 | **Mtap** | FBgn0034215 |
| Npc2g | | FBgn0039800 | **CG4995** | FBgn0032219 |
| CG11594 | | FBgn0035484 | **CG5026** | FBgn0035945 |
| GstE13 | | FBgn0033381 | **Sgt** | FBgn0032640 |
| Mesh1 | | FBgn0039650 | **CG5515** | FBgn0039163 |
| CG12279 | | FBgn0038080 | **P5cr** | FBgn0015781 |
| SmD2 | | FBgn0261789 | **spz** | FBgn0003495 |
| Hrb87F | | FBgn0004237 | **CG6180** | FBgn0032453 |
| CCHa2 | | FBgn0038147 | **CHORD** | FBgn0029503 |
| Cisd2 | | FBgn0062442 | **Bap55** | FBgn0025716 |
| CG14715 | | FBgn0037930 | **CG6805** | FBgn0034179 |
| CG1532 | | FBgn0031143 | **CG6908** | FBgn0037936 |
| CG15343 | | FBgn0030029 | **Cbp80** | FBgn0022942 |
| CG15369 | | FBgn0030105 | **LanB1** | FBgn0261800 |
| CG15717 | | FBgn0030451 | **eIF4AIII** | FBgn0037573 |
| Roc1a | | FBgn0025638 | **DNaseII** | FBgn0000477 |
| Tim8 | | FBgn0027359 | **janA** | FBgn0001280 |
| CG17737 | | FBgn0035423 | **SLIRP2** | FBgn0037602 |
| CG2004 | | FBgn0030060 | **CG8417** | FBgn0037744 |
| CG2091 | | FBgn0037372 | **CG8778** | FBgn0033761 |
| Gip | | FBgn0011770 | **mRpL24** | FBgn0031651 |
| COX7C | | FBgn0040773 | **CG9034** | FBgn0040931 |
| CG2611 | | FBgn0032871 | **Ddx1** | FBgn0015075 |
| CG2767 | | FBgn0037537 | **DENR** | FBgn0030802 |
| Cpr60D | | FBgn0050163 | **Trs23** | FBgn0260861 |
| CG30499 | | FBgn0050499 | **CG9344** | FBgn0034564 |
| CG31917 | | FBgn0031668 | **l(2)01289** | FBgn0010482 |
| CG32069 | | FBgn0052069 | **CG9667** | FBgn0037550 |
| CG3226 | | FBgn0029882 | **CG9853** | FBgn0086605 |
| CG3831 | | FBgn0034804 | **CG9914** | FBgn0030737 |
| CG3887 | | FBgn0031670 | **Cyp1** | FBgn0004432 |
| Got2 | | FBgn0001125 | **Surf1** | FBgn0029117 |
| CG4447 | | FBgn0035980 |  |  |

**Table S2:** Genes that are downregulated upon both Spen depletion in the FB and under starvation conditions [63].
