# Supplementary figures and images for: Correction: An autonomous metabolic role for Spen
Source: PLoS Genet. 2018 Mar 6;14(3):e1007266. doi: 10.1371/journal.pgen.1007266 (PMC5839538; doi:10.1371/journal.pgen.1007266)

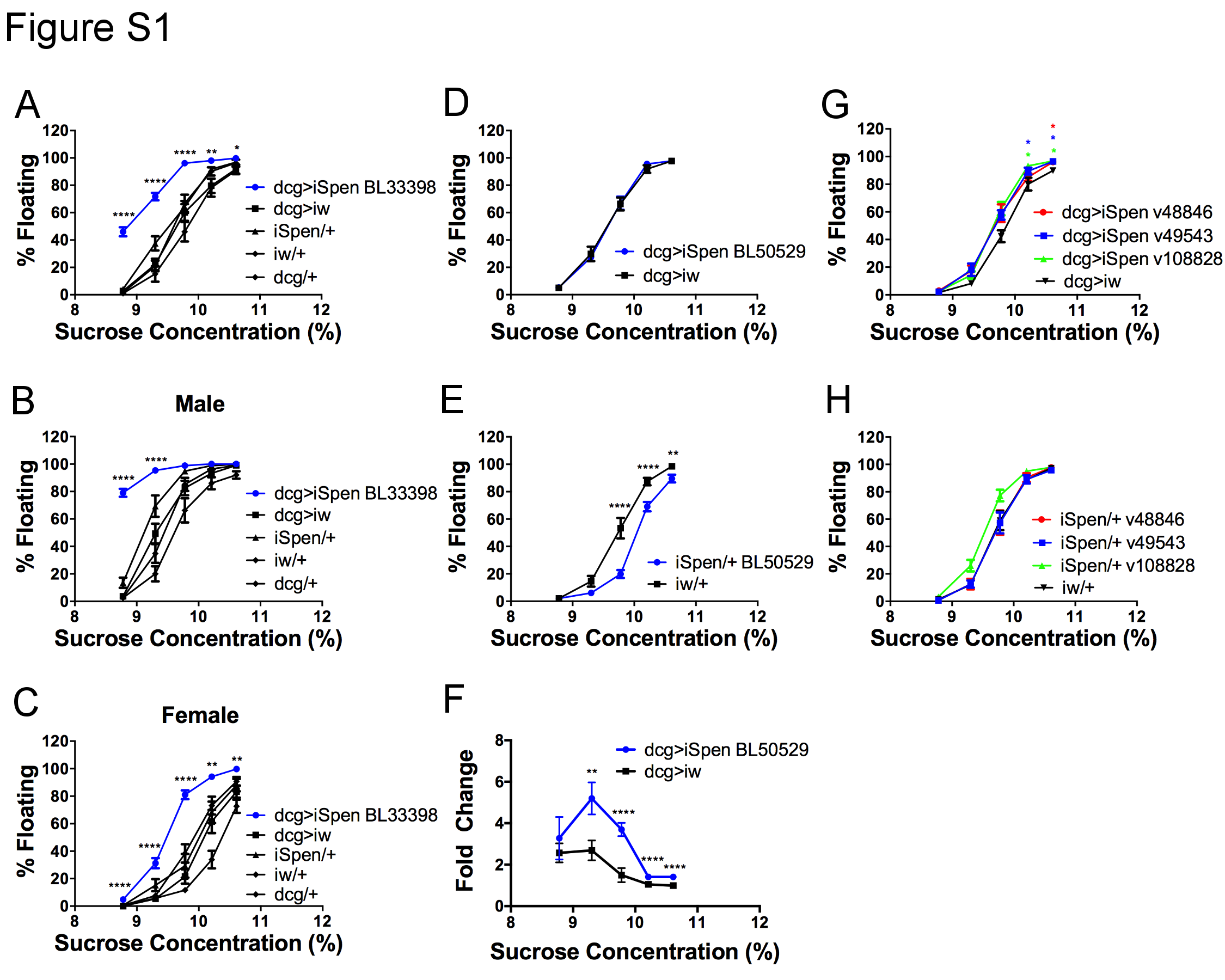

Supplement: S1 Fig — (A) Percent of floating larvae in different density solutions. FB-specific Spen KD (dcg>iSpen, BL33398) as in Fig 1A with additional dcg/+ background control. Fifty larvae per genotype per experimental replicate, n = 8 biological replicates per genotype. (B) Percent of male only Spen KD larvae floating. (C) Percent of female only Spen KD larvae floating. (D) FB-specific Spen KD (dcg>iSpen, BL50529) with different insertion site as Spen KD in Fig 1A compared to KD control (dcg>iw). (E) Genetic background controls (iSpen/+ and iw/+) for (D). (F) As the Spen hairpin insertion site appears to result in a lean phenotype, KD animals were normalized to their genetic background. (G) As in (A), three additional independent Spen hairpin constructs (dcg>iSpen) tested in different density solutions and compared to KD control (dcg>iw). (H) Genetic background controls (iSpen/+’s and iw/+) for (G). P value obtained by ANOVA. *P < 0.05, ** P < 0.01, ***P < 0.001, **** P < 0.0001. Error bars represent SEM. (TIF) [file pgen.1007266.s001.tif]
